# Supplementary figures and images for: Human DDX6 regulates translation and decay of inefficiently translated mRNAs
Source: eLife. 2024 Jul 11;13:RP92426. doi: 10.7554/eLife.92426 (PMC11239181; doi:10.7554/eLife.92426)

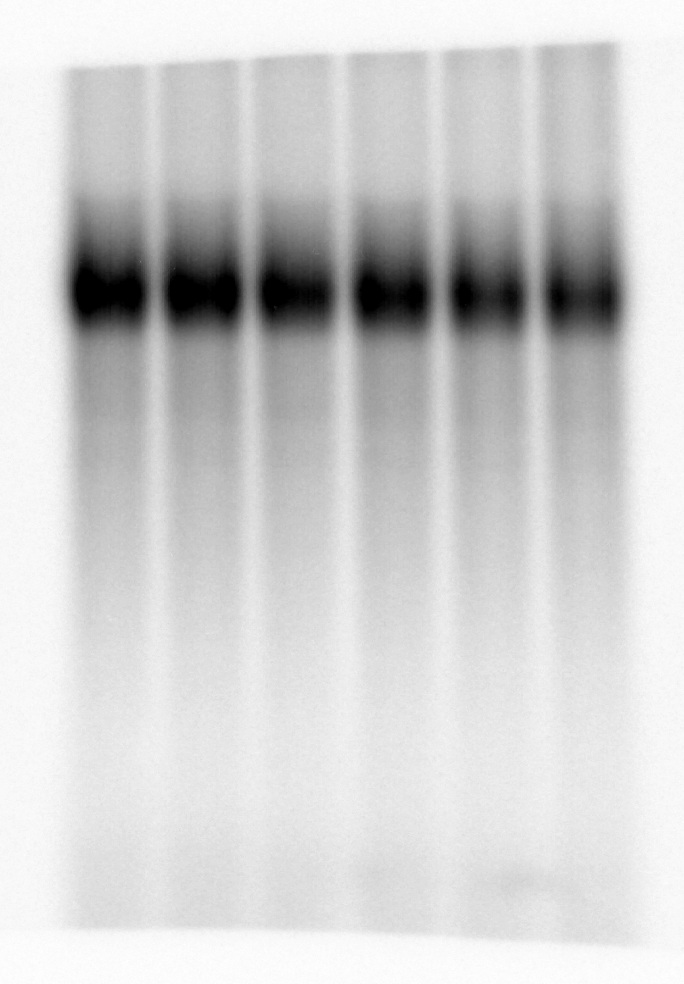

Supplement: Figure 1—source data 1. [file elife-92426-fig1-data1.zip › Figure 1-source data 1_Original file for the Northern blot analysis in Figure 1B/DDX6ko-30RC.tif]

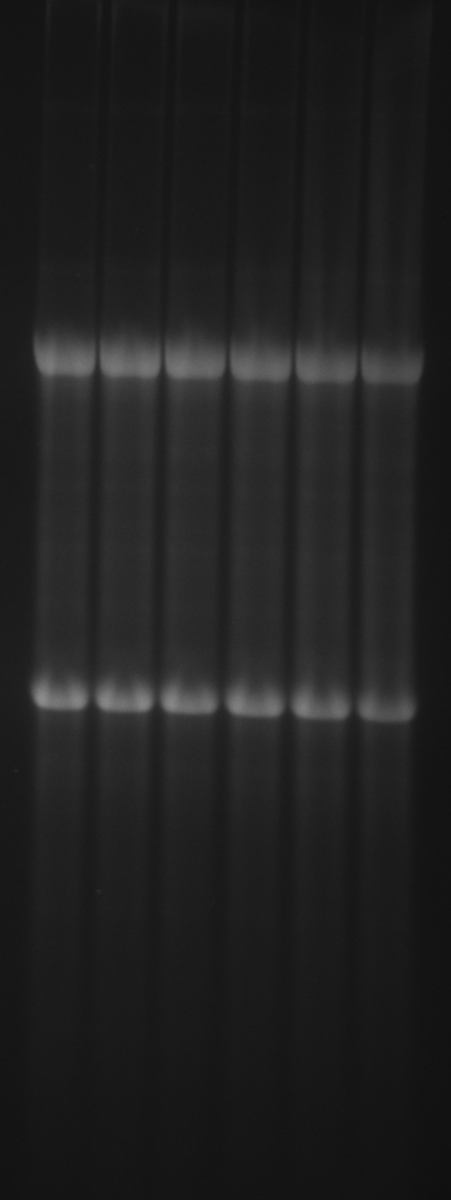

Supplement: Figure 1—source data 1. [file elife-92426-fig1-data1.zip › Figure 1-source data 1_Original file for the Northern blot analysis in Figure 1B/DDX6ko-30RC_ribo.tif]

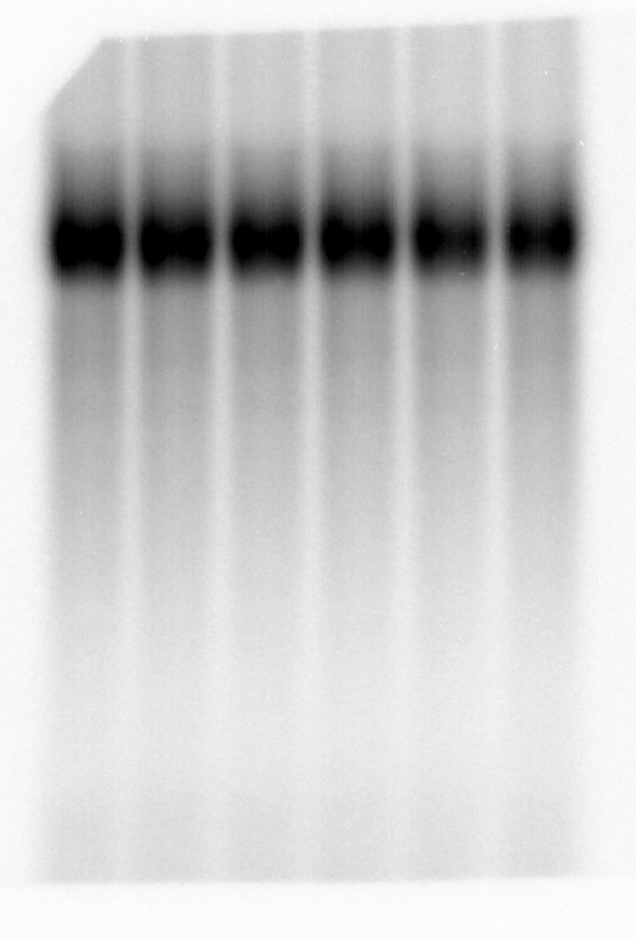

Supplement: Figure 1—source data 1. [file elife-92426-fig1-data1.zip › Figure 1-source data 1_Original file for the Northern blot analysis in Figure 1B/DDX6ko-ctrl.tif]

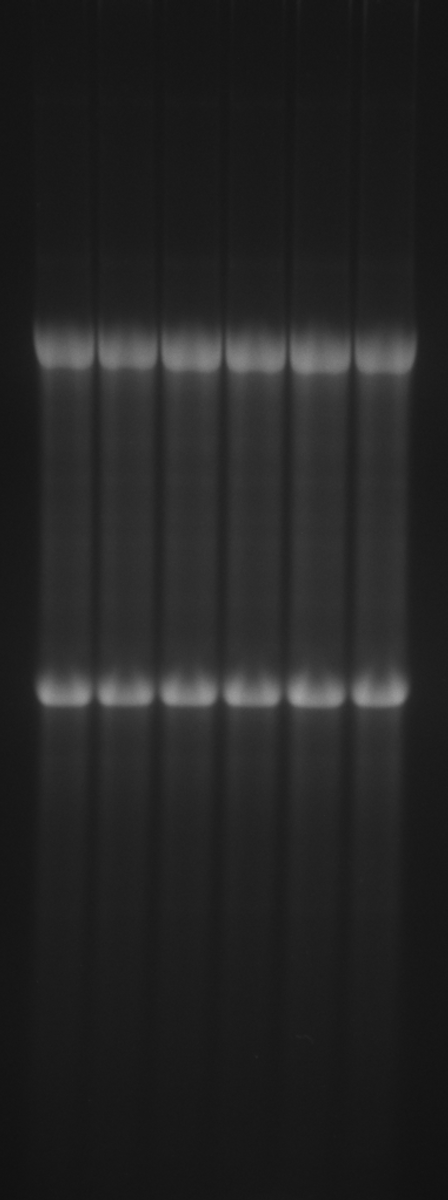

Supplement: Figure 1—source data 1. [file elife-92426-fig1-data1.zip › Figure 1-source data 1_Original file for the Northern blot analysis in Figure 1B/DDX6ko_ctrl_ribo.tif]

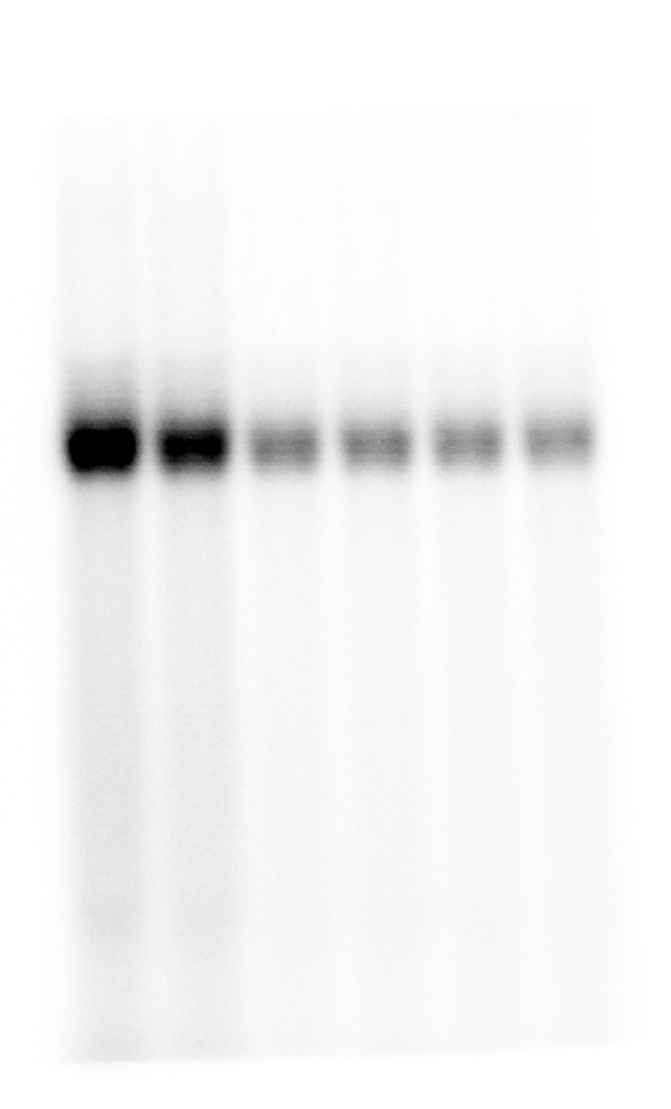

Supplement: Figure 1—source data 1. [file elife-92426-fig1-data1.zip › Figure 1-source data 1_Original file for the Northern blot analysis in Figure 1B/WT-30RC.tif]

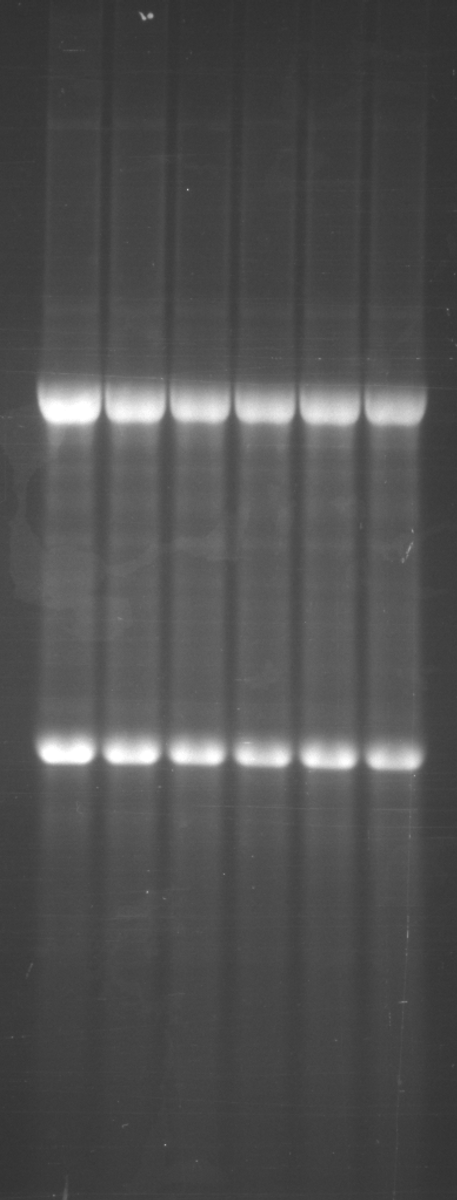

Supplement: Figure 1—source data 1. [file elife-92426-fig1-data1.zip › Figure 1-source data 1_Original file for the Northern blot analysis in Figure 1B/WT-30RC_ribo.tif]

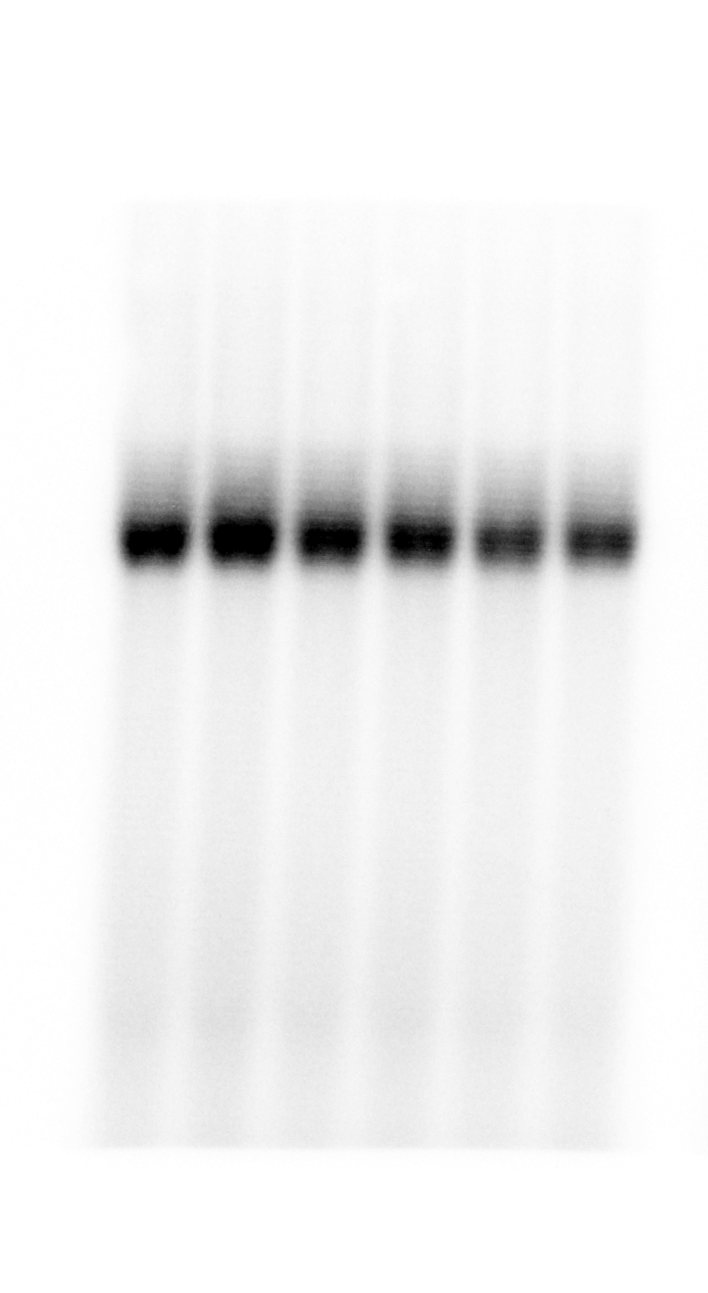

Supplement: Figure 1—source data 1. [file elife-92426-fig1-data1.zip › Figure 1-source data 1_Original file for the Northern blot analysis in Figure 1B/WT-Ctrl.tif]

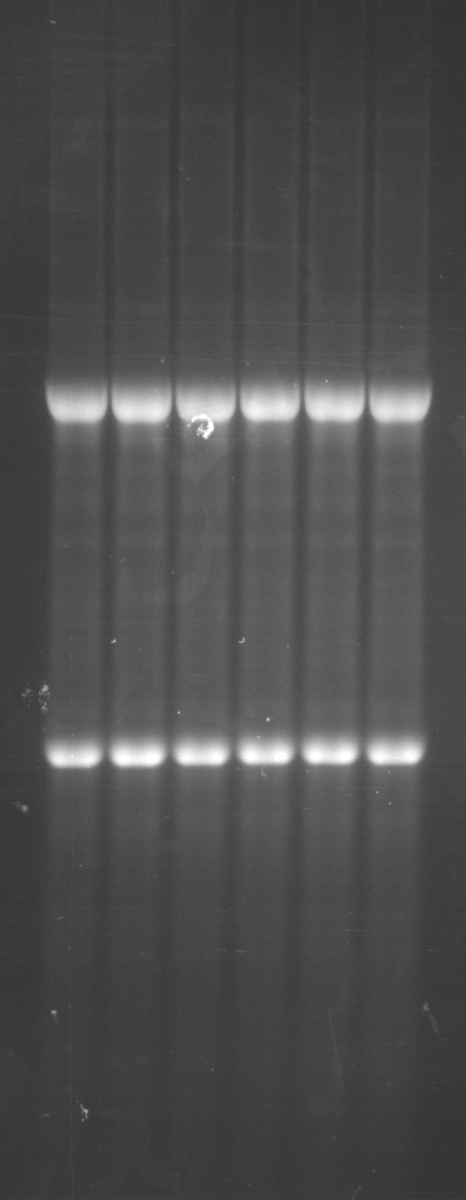

Supplement: Figure 1—source data 1. [file elife-92426-fig1-data1.zip › Figure 1-source data 1_Original file for the Northern blot analysis in Figure 1B/WT-Ctrl_ribo.tif]

Figure 1

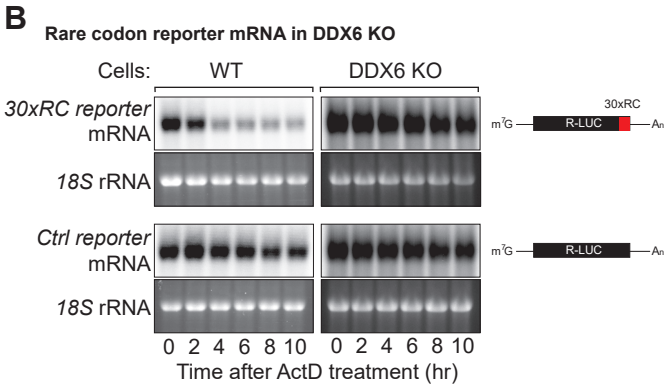

**B** Rare codon reporter mRNA in DDX6 KO

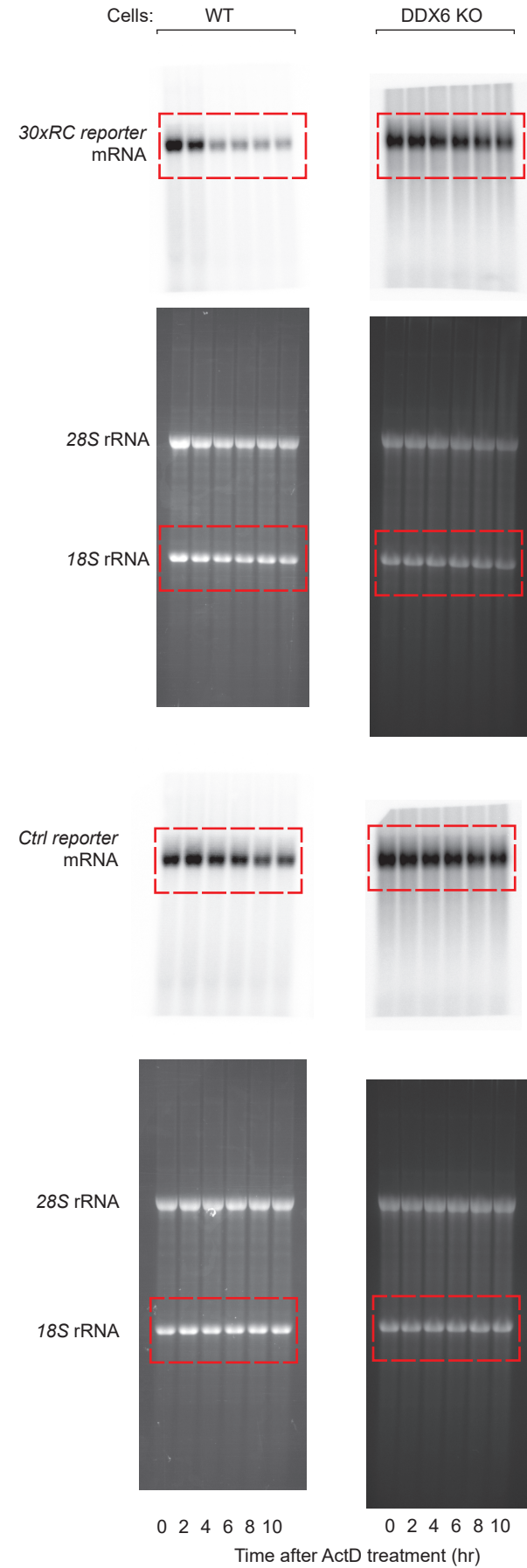

Supplement: Figure 1—source data 2. [file elife-92426-fig1-data2.pdf]

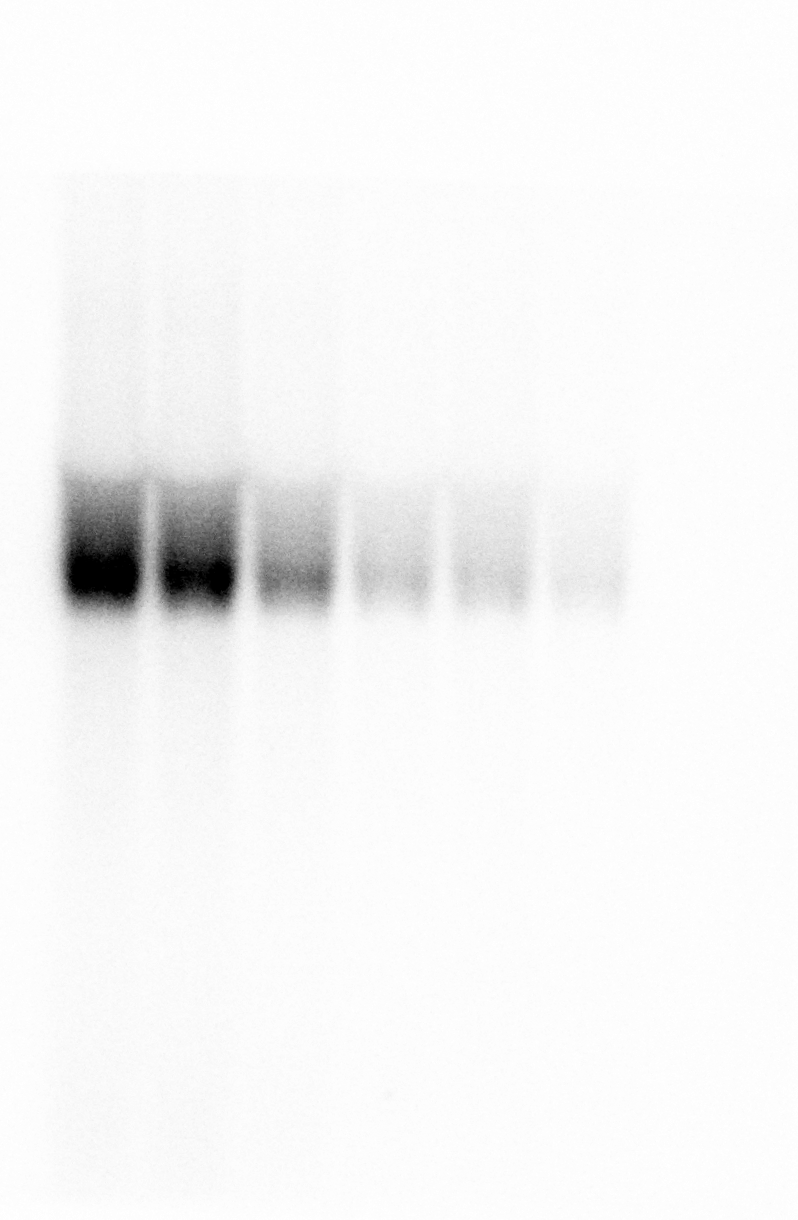

Supplement: Figure 1—source data 3. [file elife-92426-fig1-data3.zip › Figure 1-source data 3_Original file for the Northern blot analysis in Figure 1D/20180703_WT+MBP-30RC.tif]

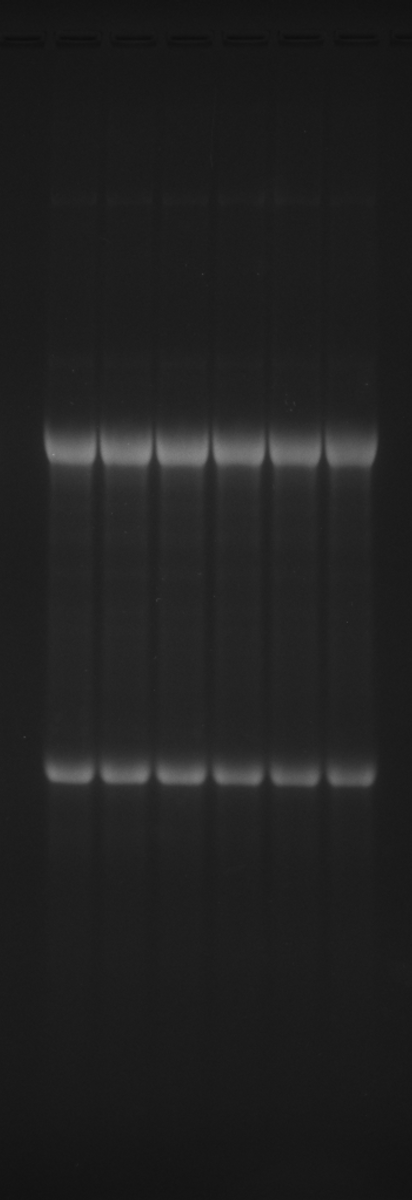

Supplement: Figure 1—source data 3. [file elife-92426-fig1-data3.zip › Figure 1-source data 3_Original file for the Northern blot analysis in Figure 1D/20180703_WT+MBP-30RC_ribo.tif]

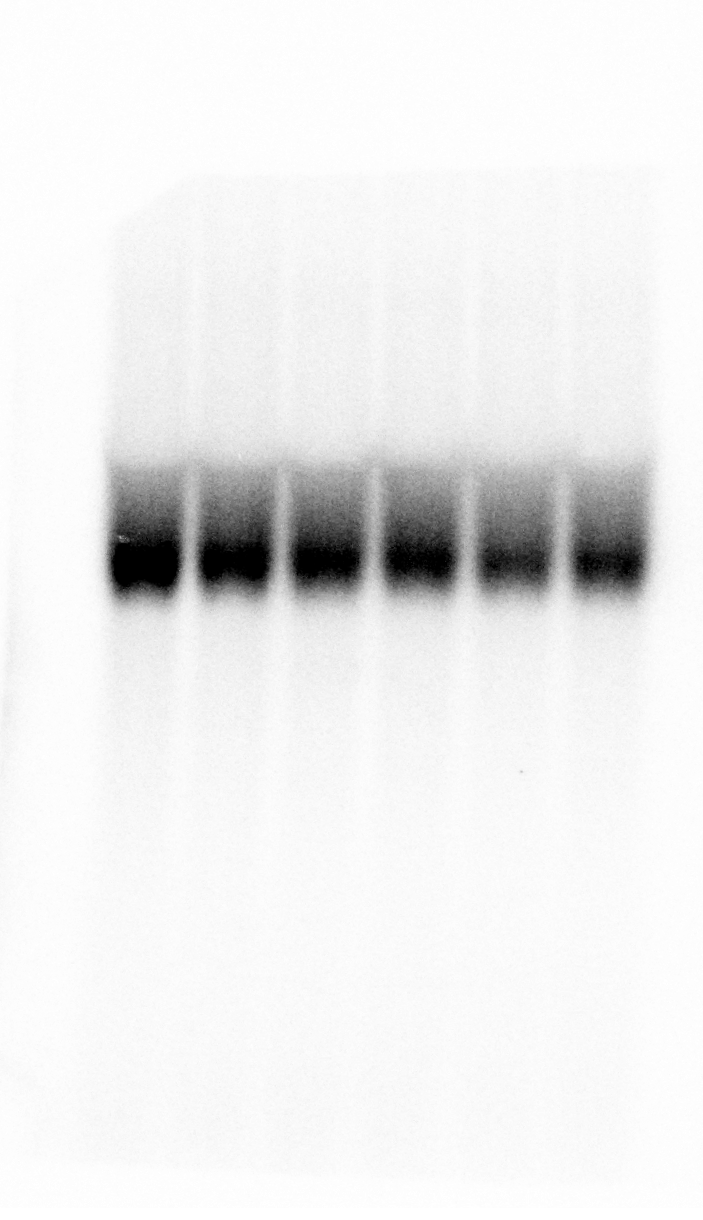

Supplement: Figure 1—source data 3. [file elife-92426-fig1-data3.zip › Figure 1-source data 3_Original file for the Northern blot analysis in Figure 1D/WT+MBP-Ctrl.tif]

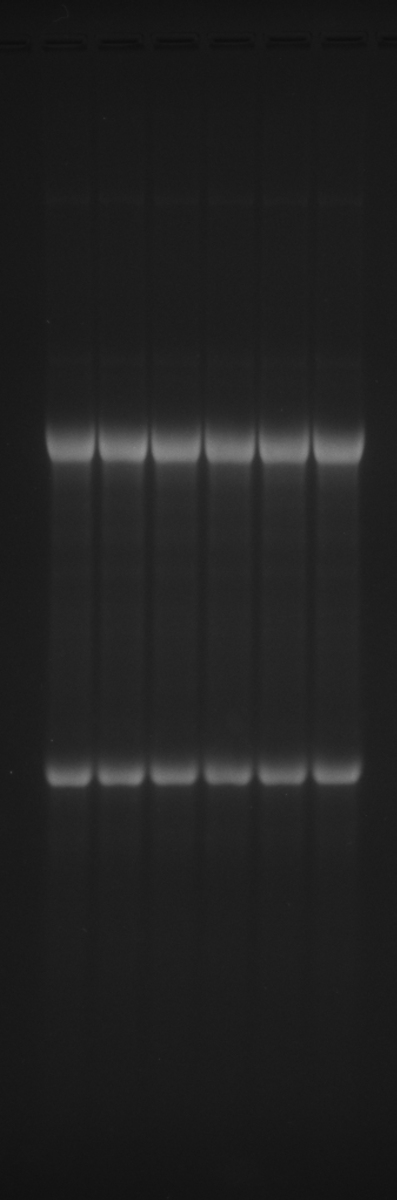

Supplement: Figure 1—source data 3. [file elife-92426-fig1-data3.zip › Figure 1-source data 3_Original file for the Northern blot analysis in Figure 1D/WT+MBP-Ctrl_ribo.tif]

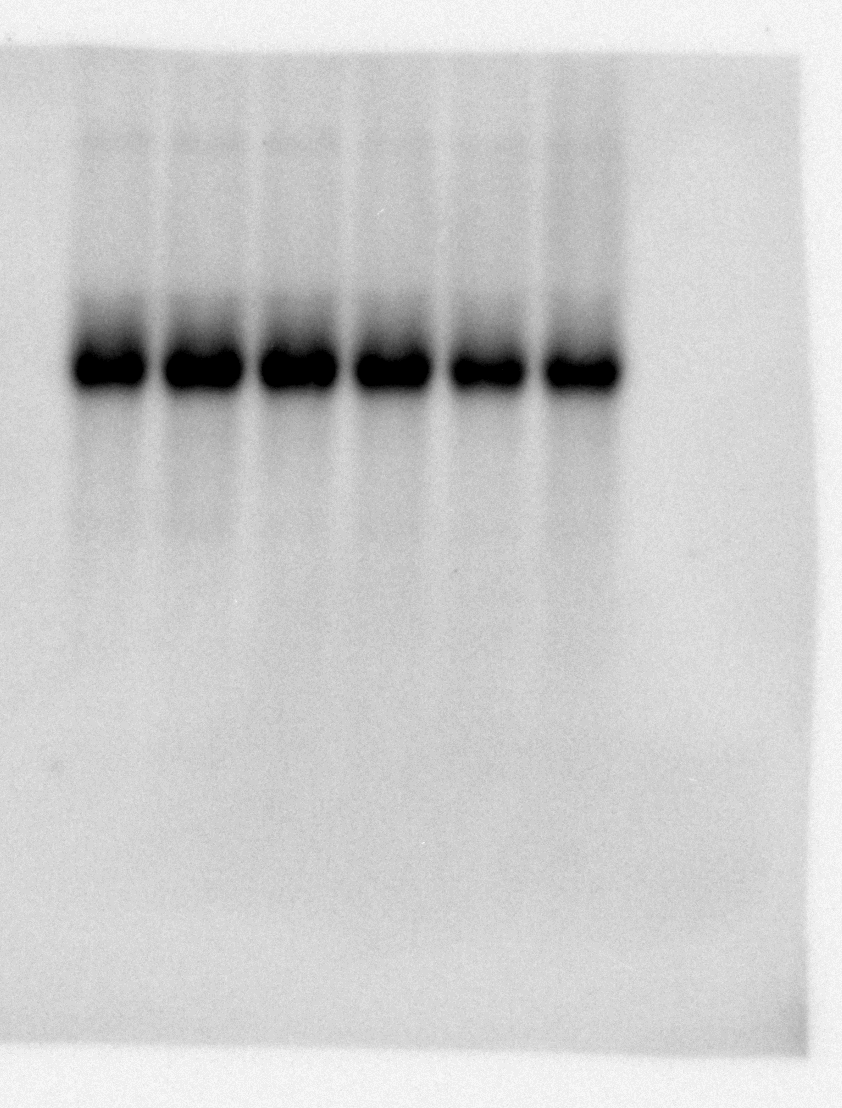

Supplement: Figure 1—source data 3. [file elife-92426-fig1-data3.zip › Figure 1-source data 3_Original file for the Northern blot analysis in Figure 1D/WT+POP2mut-30RC.tif]

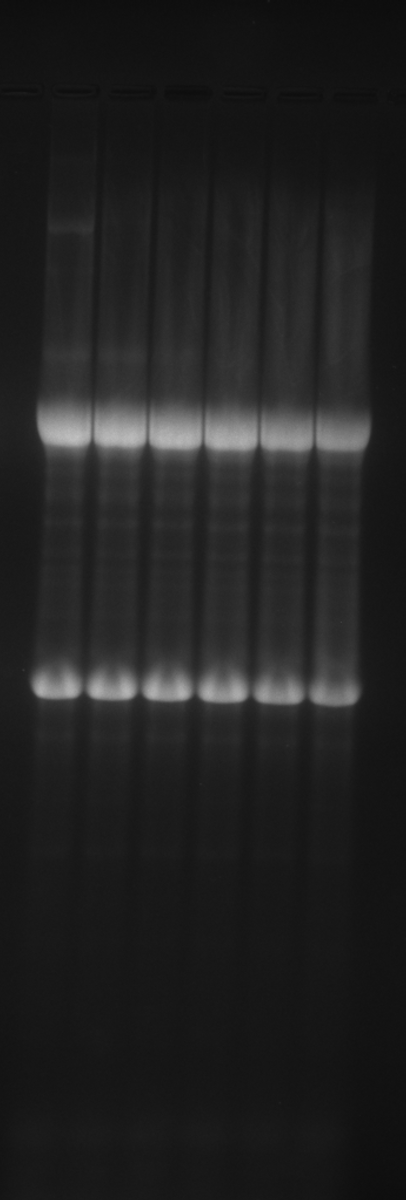

Supplement: Figure 1—source data 3. [file elife-92426-fig1-data3.zip › Figure 1-source data 3_Original file for the Northern blot analysis in Figure 1D/WT+POP2mut-30RC_ribo.tif]

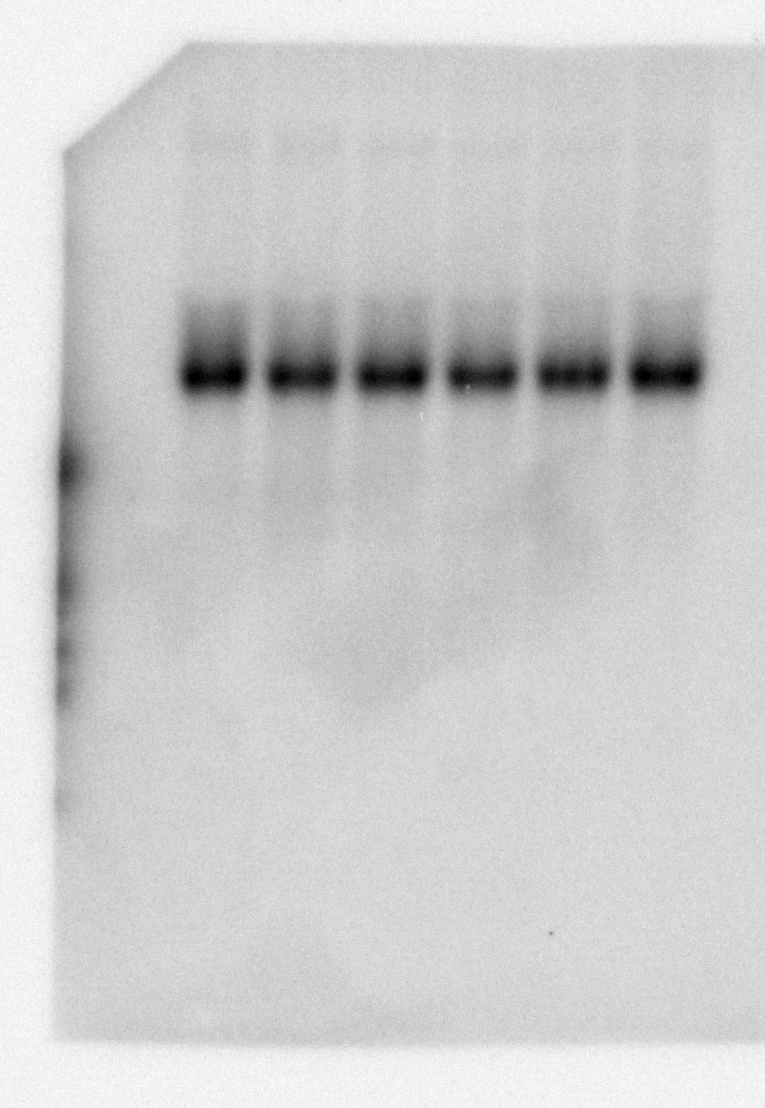

Supplement: Figure 1—source data 3. [file elife-92426-fig1-data3.zip › Figure 1-source data 3_Original file for the Northern blot analysis in Figure 1D/WT+POP2mut-Ctrl.tif]

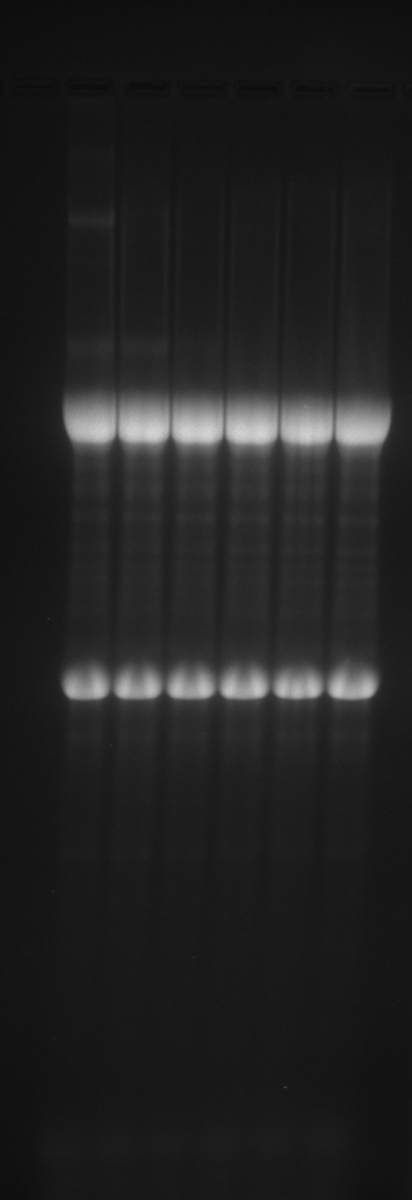

Supplement: Figure 1—source data 3. [file elife-92426-fig1-data3.zip › Figure 1-source data 3_Original file for the Northern blot analysis in Figure 1D/WT+POP2mut-Ctrl_ribo.tif]

Figure 1

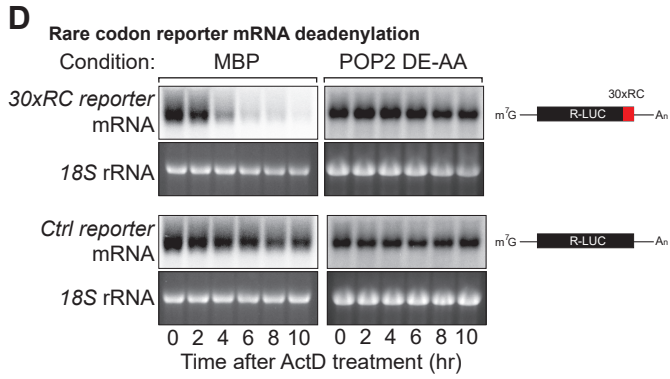

**D** Rare codon reporter mRNA deadenylation

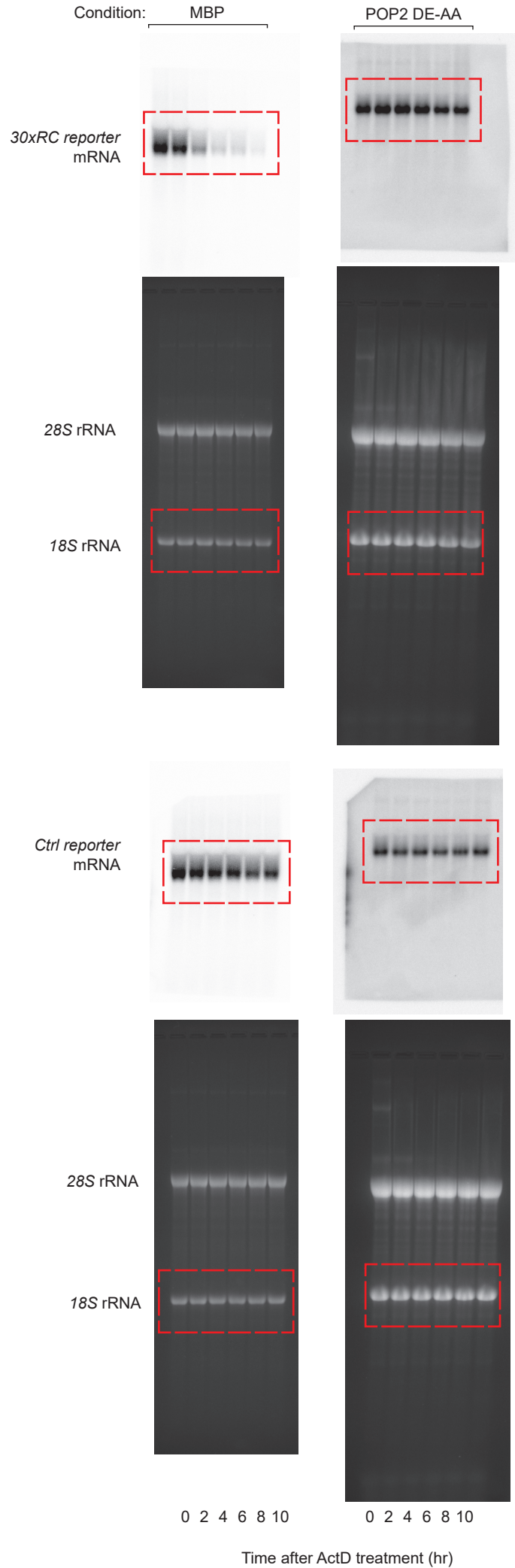

Supplement: Figure 1—source data 4. [file elife-92426-fig1-data4.pdf]

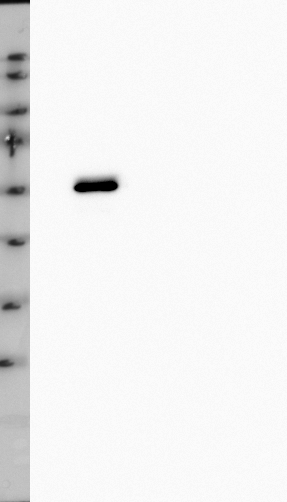

Supplement: Figure 1—figure supplement 1—source data 1. [file elife-92426-fig1-figsupp1-data1.zip › Figure 1-figure supplement 1-Source Data 1_Original file for the Western Blot analysis in Figure 1-figure supplement 1A/aDDX6.tif]

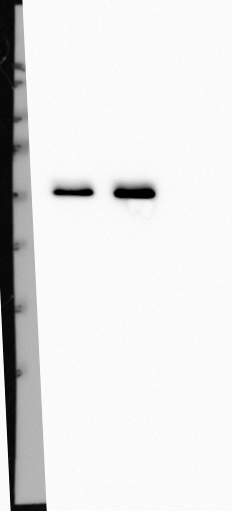

Supplement: Figure 1—figure supplement 1—source data 1. [file elife-92426-fig1-figsupp1-data1.zip › Figure 1-figure supplement 1-Source Data 1_Original file for the Western Blot analysis in Figure 1-figure supplement 1A/atubulin.tif]

Figure 1-figure supplement 1

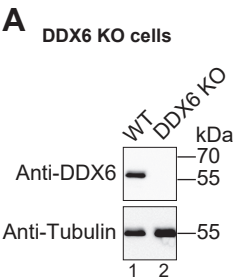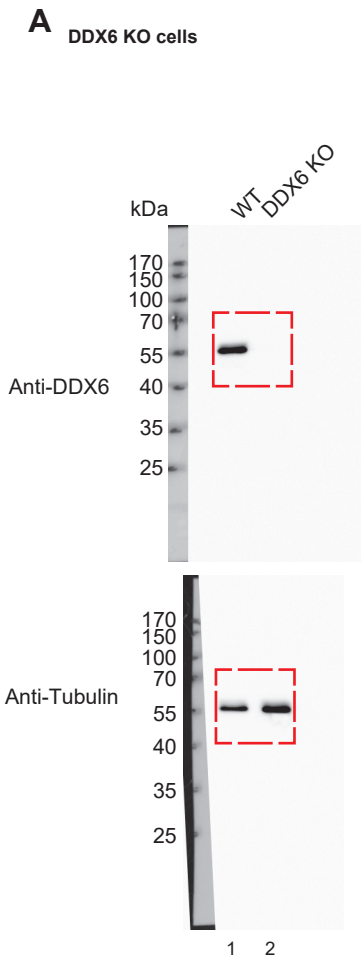

Supplement: Figure 1—figure supplement 1—source data 2. [file elife-92426-fig1-figsupp1-data2.pdf]

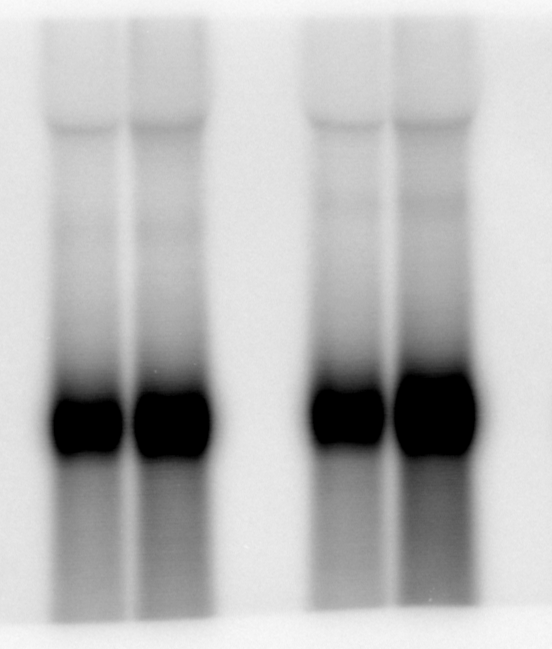

Supplement: Figure 1—figure supplement 1—source data 3. [file elife-92426-fig1-figsupp1-data3.zip › Figure 1-figure supplement 1-Source Data 3_Original file for the Northern blot analysis in Figure 1-figure supplement 1E/FLUC.tif]

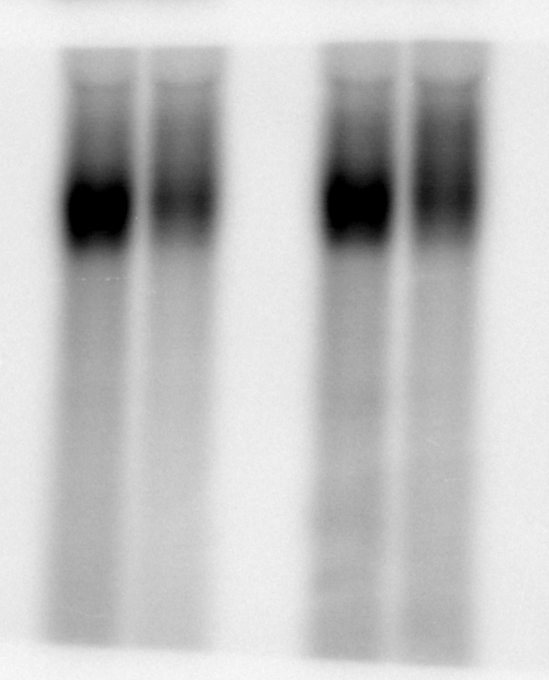

Supplement: Figure 1—figure supplement 1—source data 3. [file elife-92426-fig1-figsupp1-data3.zip › Figure 1-figure supplement 1-Source Data 3_Original file for the Northern blot analysis in Figure 1-figure supplement 1E/RLUC.tif]

Figure 1-figure supplement 1

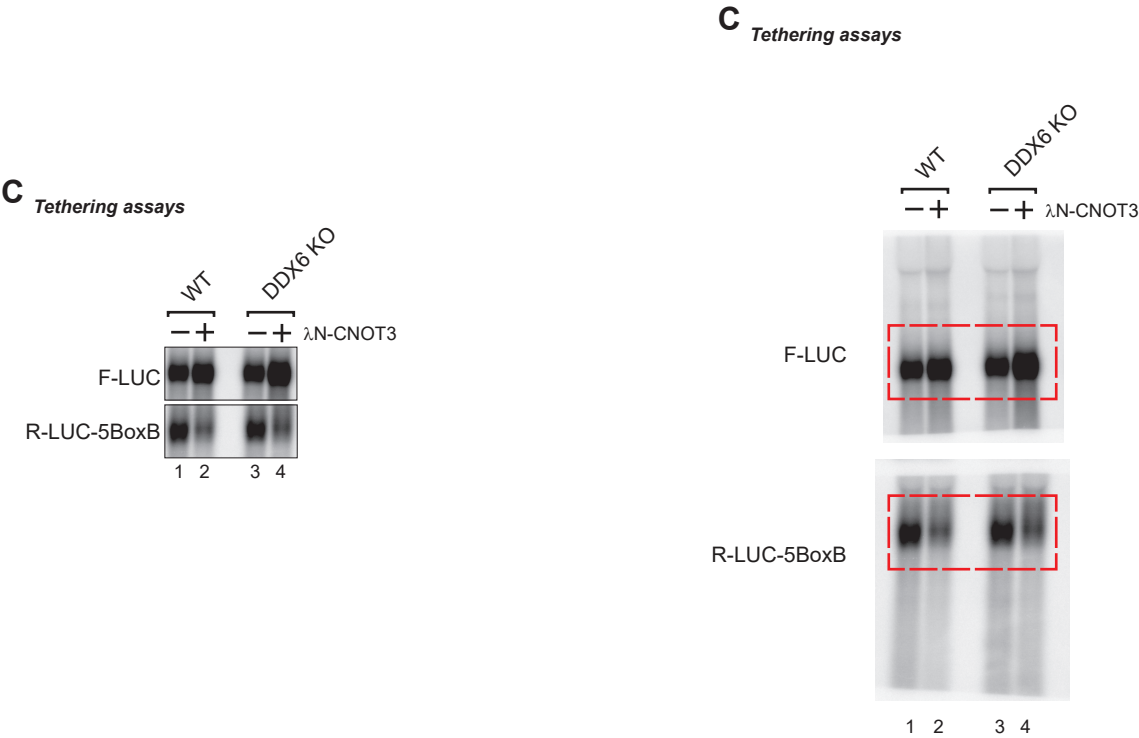

Supplement: Figure 1—figure supplement 1—source data 4. [file elife-92426-fig1-figsupp1-data4.pdf]

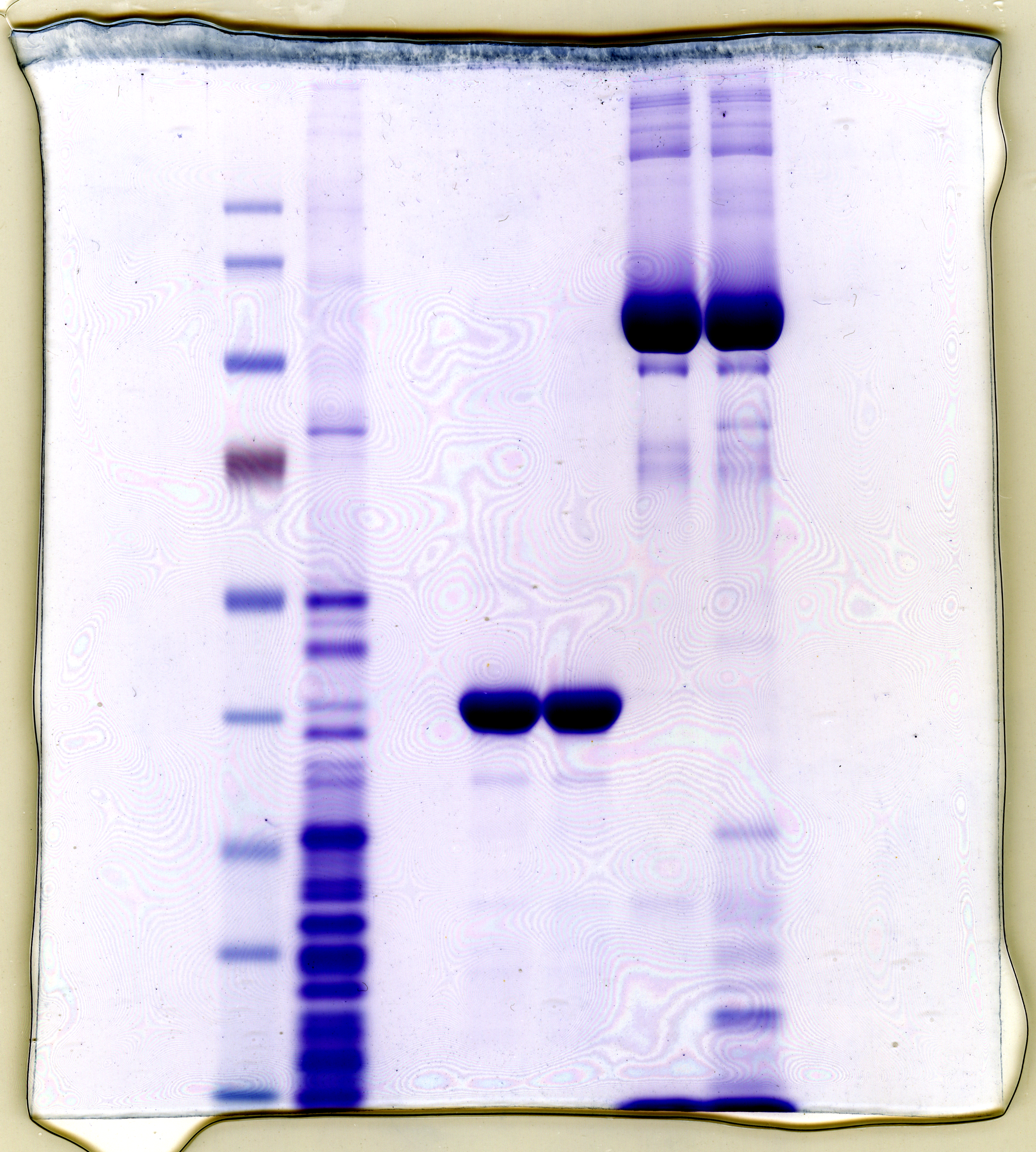

Supplement: Figure 2—source data 1. [file elife-92426-fig2-data1.zip › Figure 2-source data 1_Original file for the SDS-PAGE in Figure 2A/DDX6 pulldown ribo.tif]

Figure 2

**A** DDX6 – Ribosome interaction *in vitro*

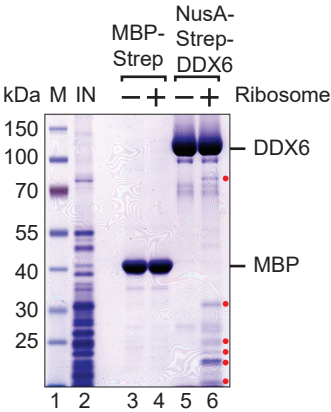

**A** DDX6 – Ribosome interaction *in vitro*

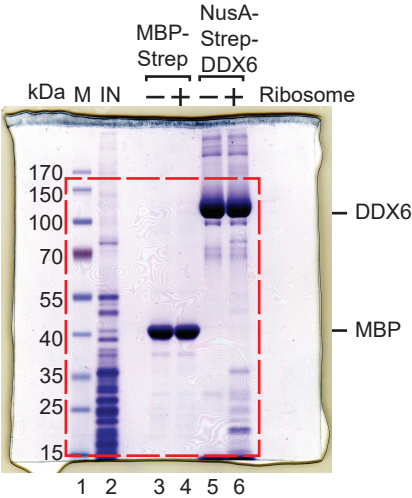

Supplement: Figure 2—source data 2. [file elife-92426-fig2-data2.pdf]

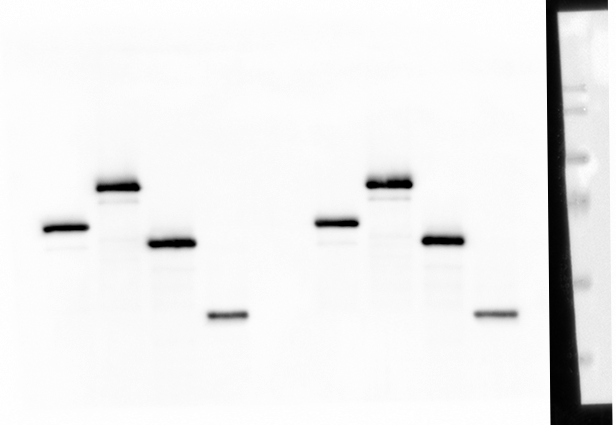

Supplement: Figure 2—source data 3. [file elife-92426-fig2-data3.zip › Figure 2-source data 3_Original file for the Western blot in Figure 2B/aGFP-DDX6.tif]

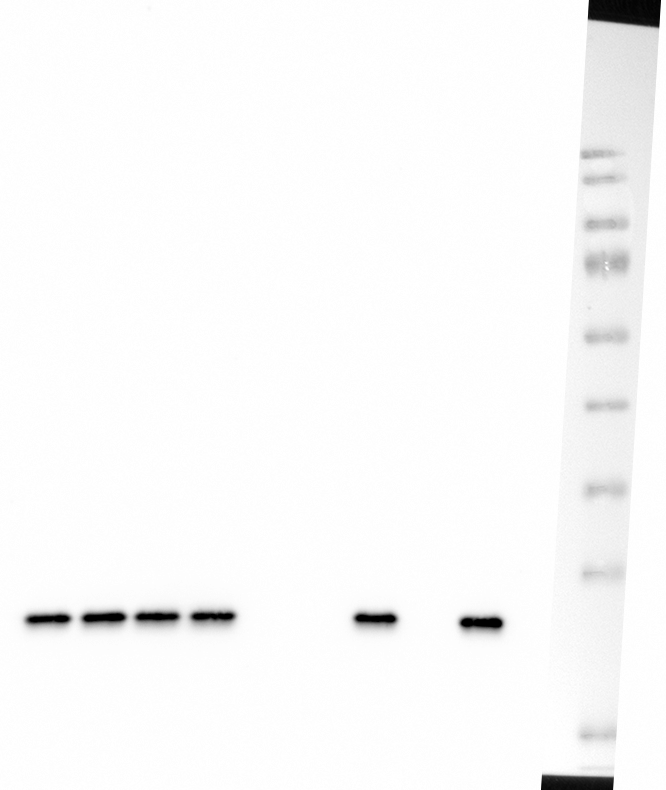

Supplement: Figure 2—source data 3. [file elife-92426-fig2-data3.zip › Figure 2-source data 3_Original file for the Western blot in Figure 2B/aHA-RPL22.tif]

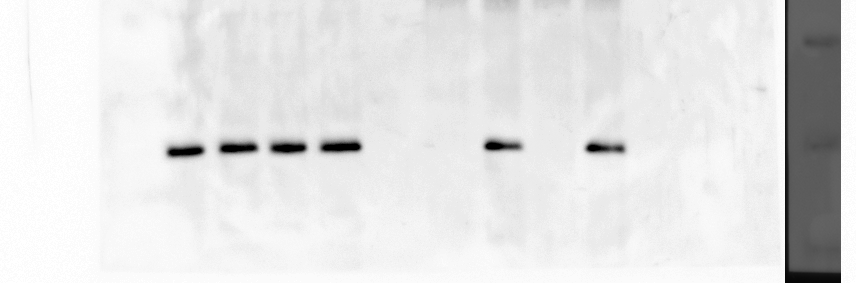

Supplement: Figure 2—source data 3. [file elife-92426-fig2-data3.zip › Figure 2-source data 3_Original file for the Western blot in Figure 2B/aRPS3A.tif]

Figure 2

**B** DDX6 – Ribosome interaction in cells

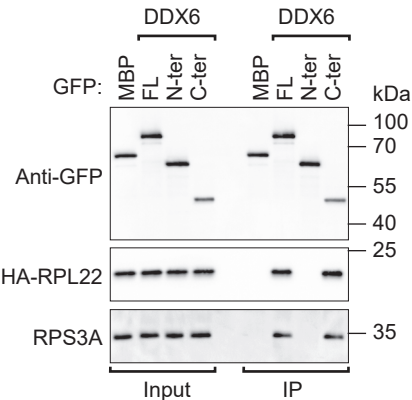

**B** DDX6 – Ribosome interaction in cells

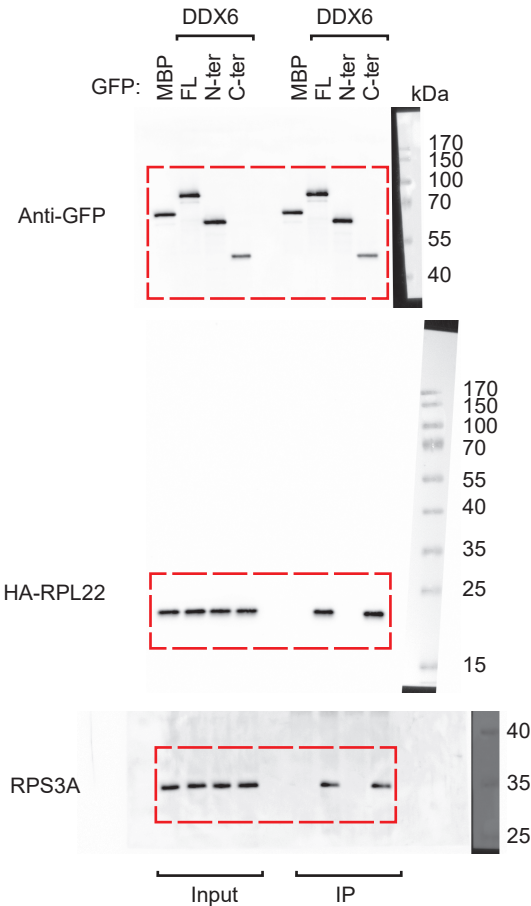

Supplement: Figure 2—source data 4. [file elife-92426-fig2-data4.pdf]

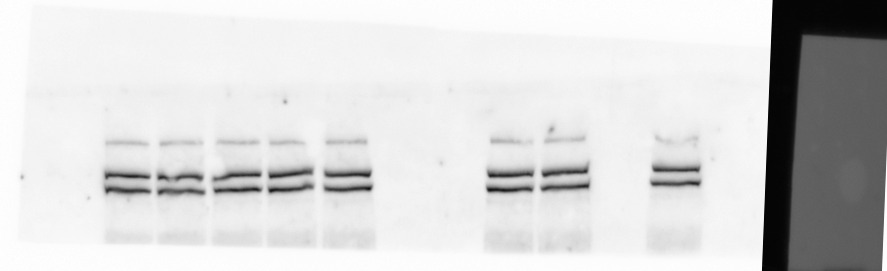

Supplement: Figure 2—source data 5. [file elife-92426-fig2-data5.zip › Figure 2-source data 5_Original file for the Western blot in Figure 2C/aCNOT1.tif]

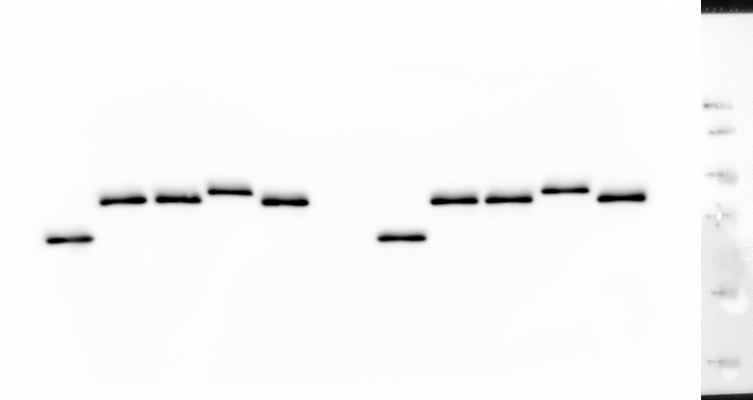

Supplement: Figure 2—source data 5. [file elife-92426-fig2-data5.zip › Figure 2-source data 5_Original file for the Western blot in Figure 2C/aGFP-DDX6 muts.tif]

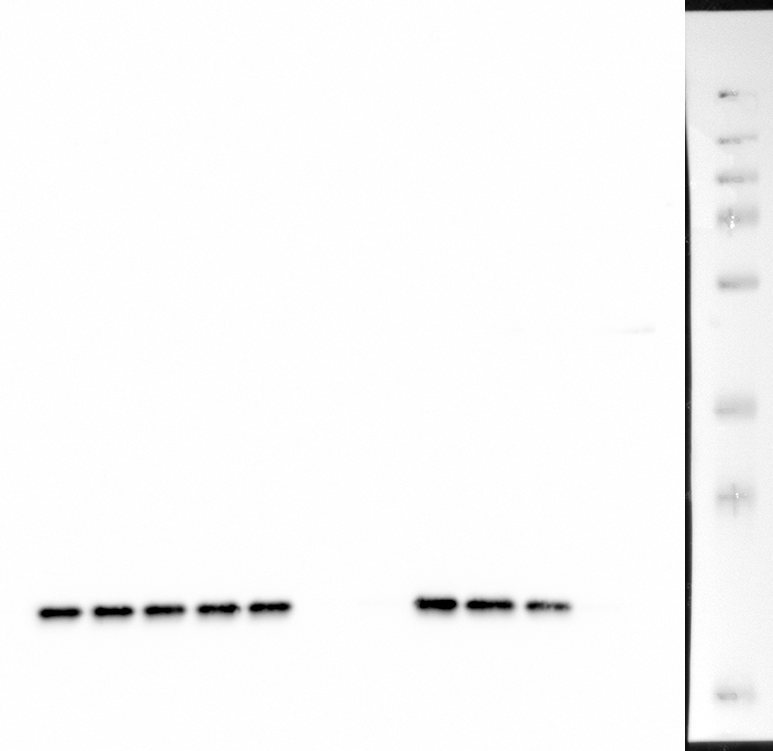

Supplement: Figure 2—source data 5. [file elife-92426-fig2-data5.zip › Figure 2-source data 5_Original file for the Western blot in Figure 2C/aHA-RPL22.tif]

Figure 2

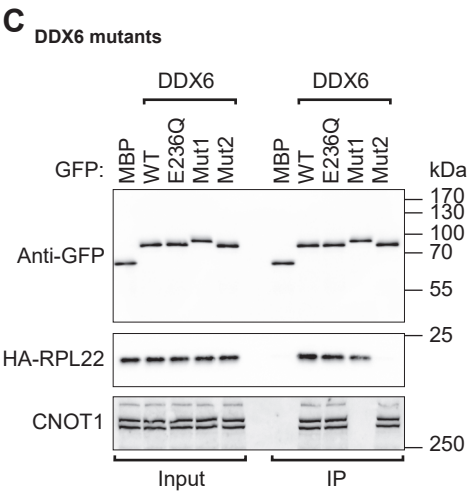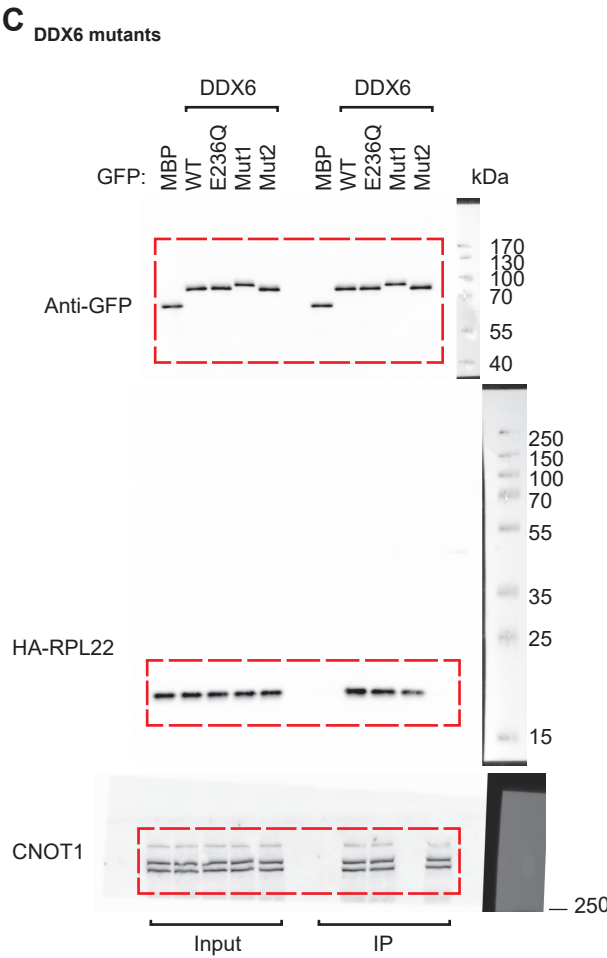

Supplement: Figure 2—source data 6. [file elife-92426-fig2-data6.pdf]

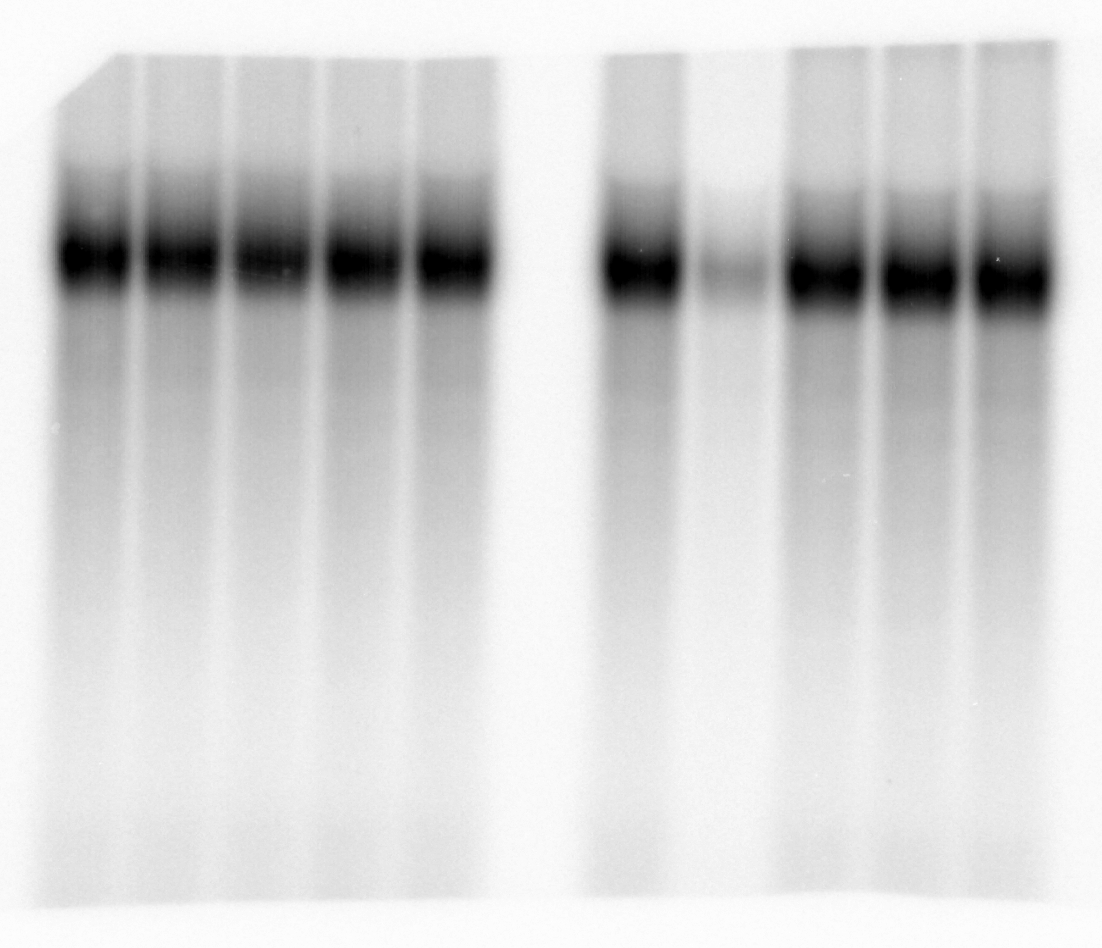

Supplement: Figure 2—source data 7. [file elife-92426-fig2-data7.zip › Figure 2-source data 7_Original file for the Northern blot in Figure 2D/DDX6 complement.tif]

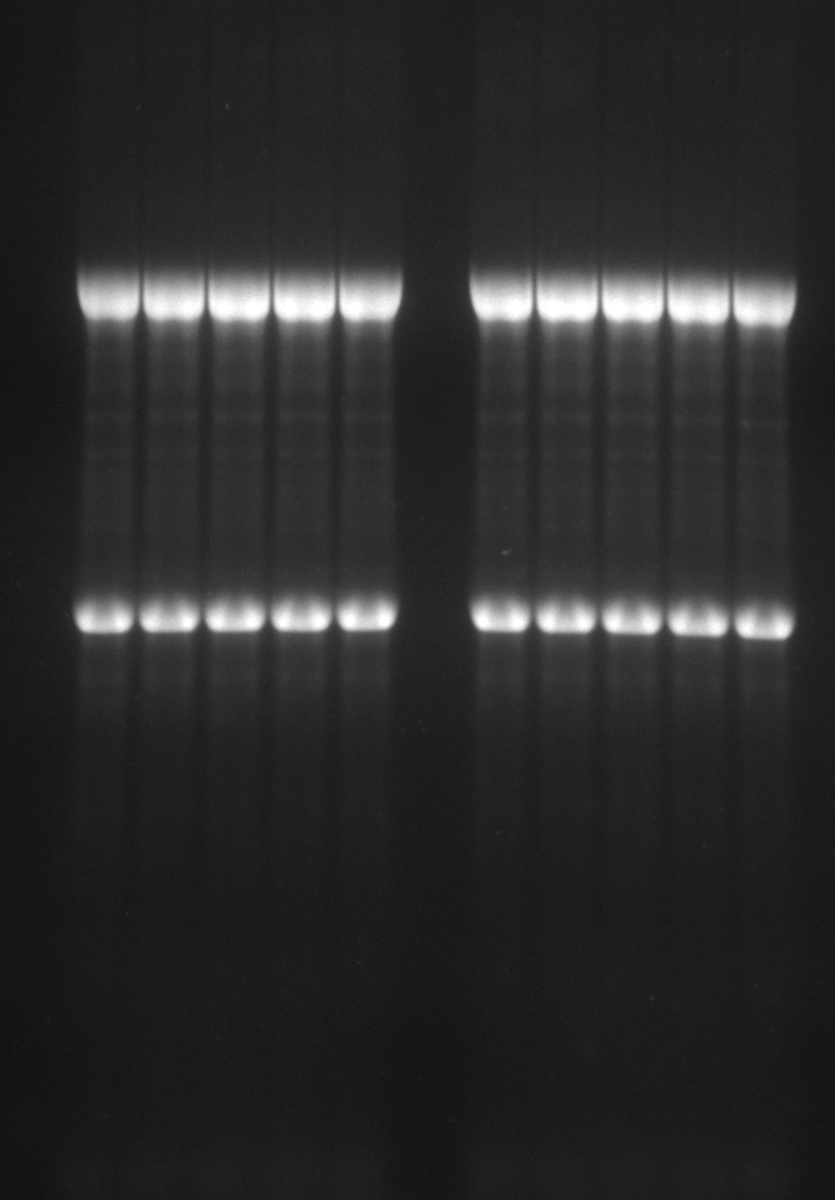

Supplement: Figure 2—source data 7. [file elife-92426-fig2-data7.zip › Figure 2-source data 7_Original file for the Northern blot in Figure 2D/DDX6 complement_ribo.tif]

Figure 2

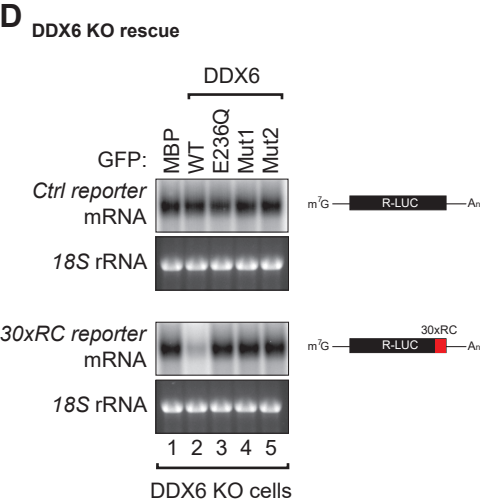

**D** DDX6 KO rescue

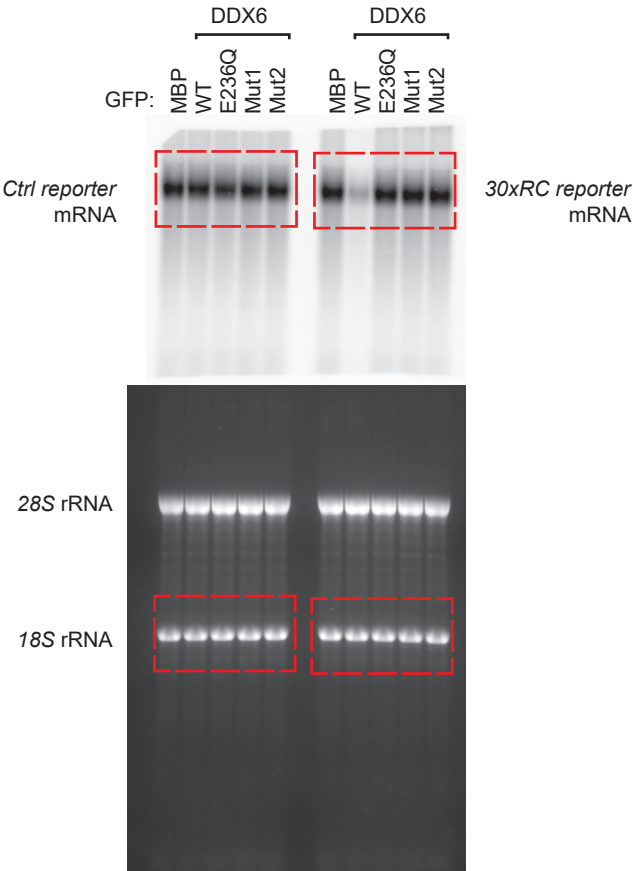

Supplement: Figure 2—source data 8. [file elife-92426-fig2-data8.pdf]

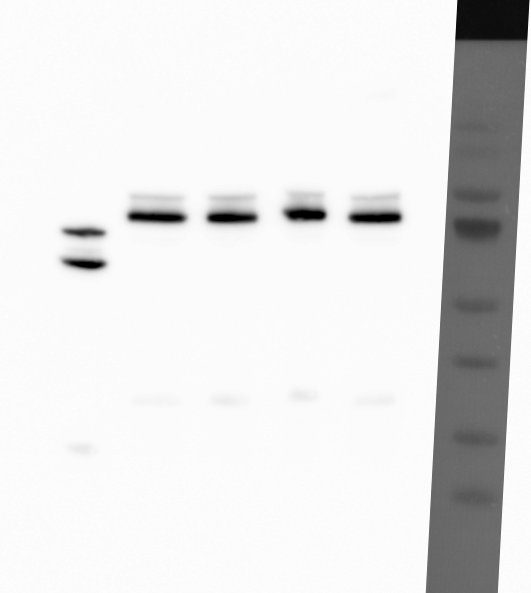

Supplement: Figure 2—source data 9. [file elife-92426-fig2-data9.zip › Figure 2-source data 9_Original file for the Westhern blot in Figure 2F/aGFP-DDX6.tif]

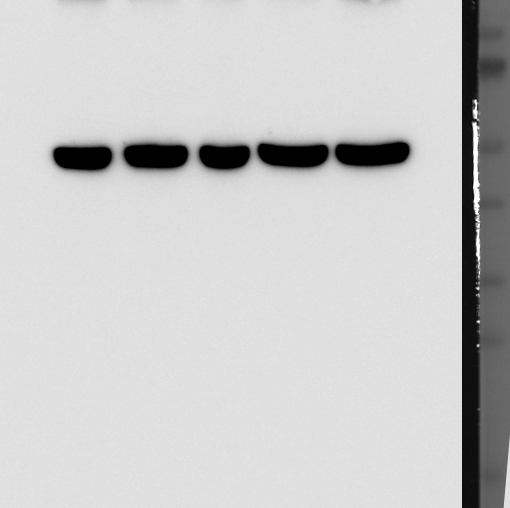

Supplement: Figure 2—source data 9. [file elife-92426-fig2-data9.zip › Figure 2-source data 9_Original file for the Westhern blot in Figure 2F/atubulin_1m.tif]

Figure 2

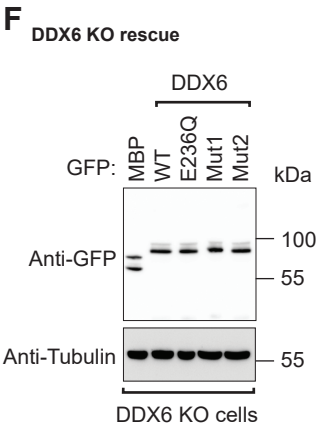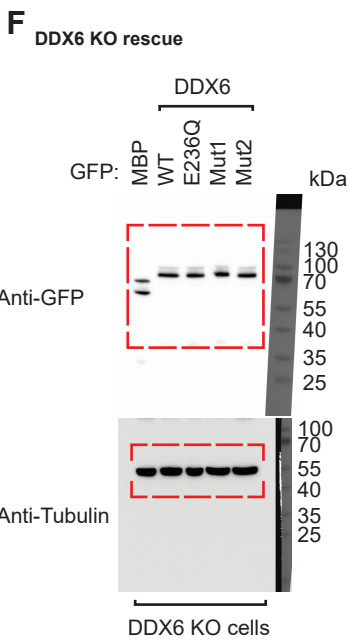

Supplement: Figure 2—source data 10. [file elife-92426-fig2-data10.pdf]

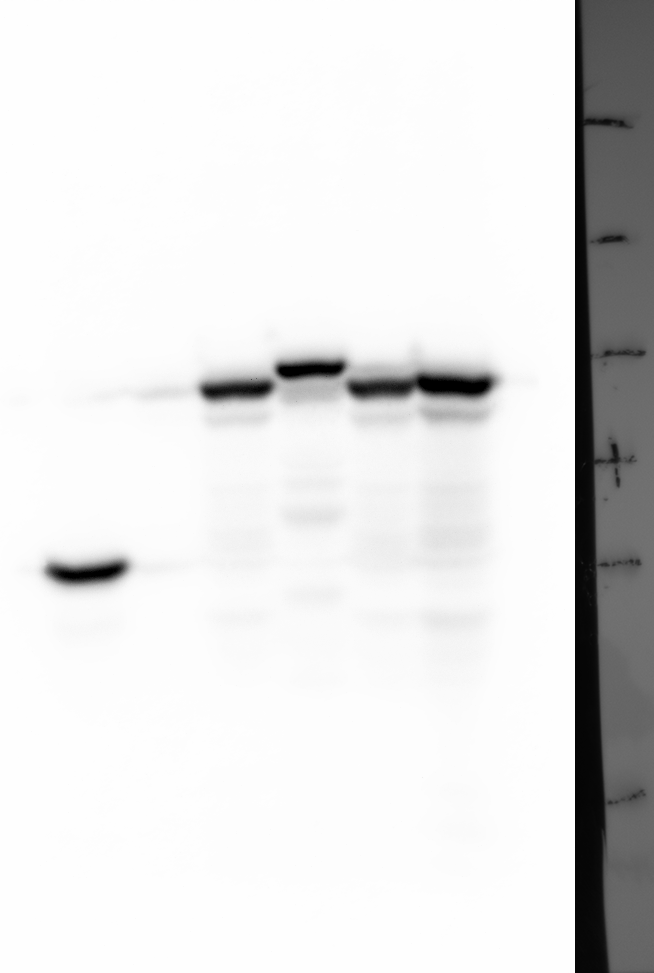

Supplement: Figure 3—source data 1. [file elife-92426-fig3-data1.zip › Figure 3-source data 1_Original file for the Westhern blot in Figure 3I/aDDX6.tif]

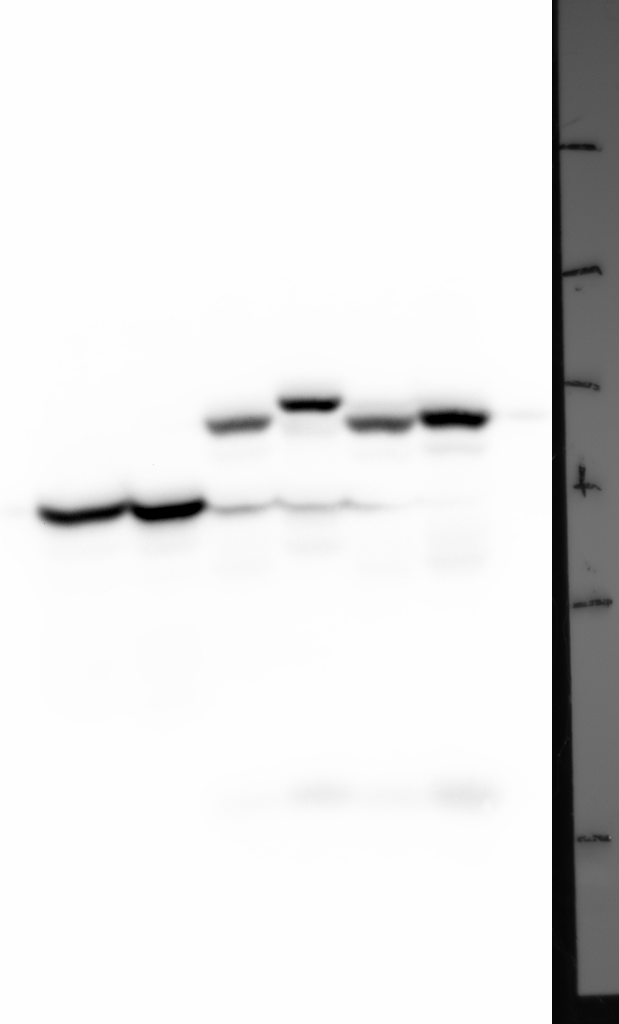

Supplement: Figure 3—source data 1. [file elife-92426-fig3-data1.zip › Figure 3-source data 1_Original file for the Westhern blot in Figure 3I/aGFP-DDX6.tif]

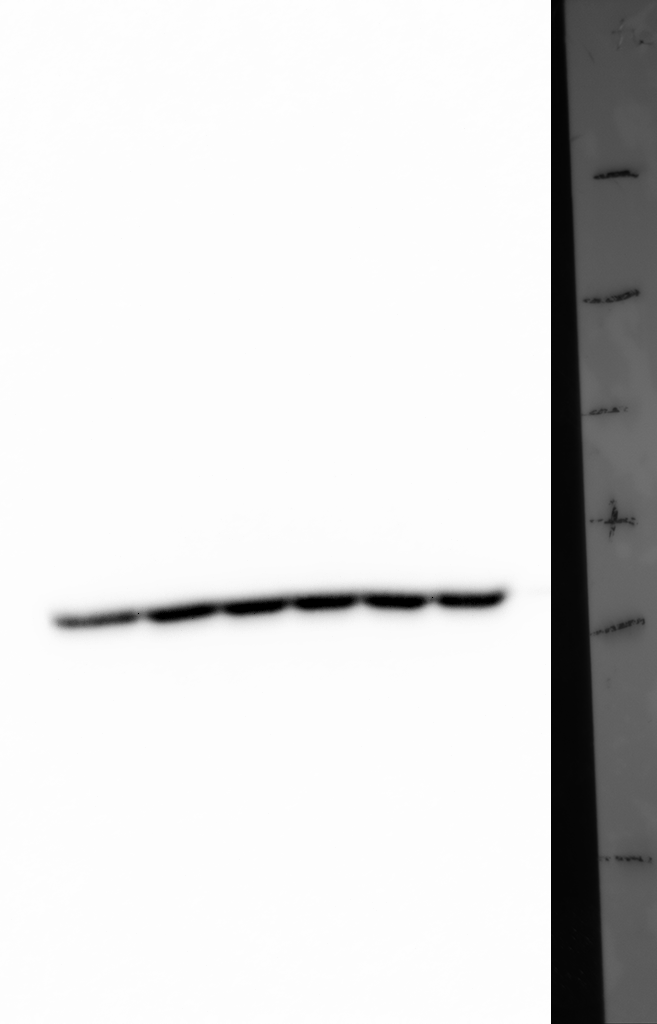

Supplement: Figure 3—source data 1. [file elife-92426-fig3-data1.zip › Figure 3-source data 1_Original file for the Westhern blot in Figure 3I/atubulin.tif]

Figure 3

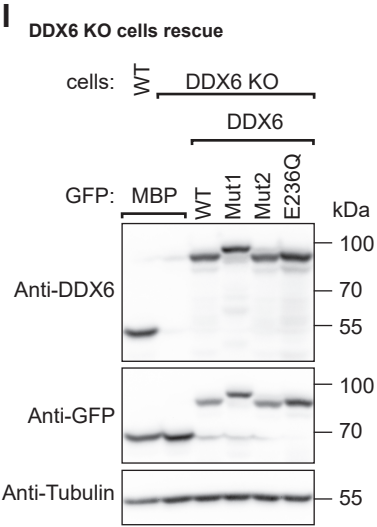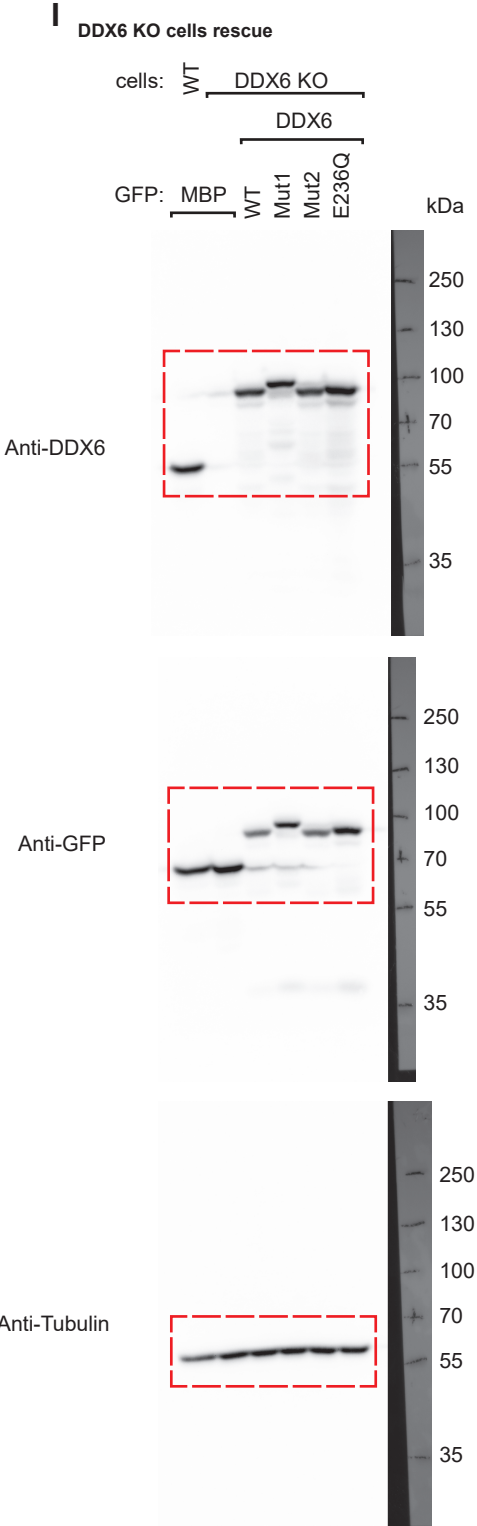

Supplement: Figure 3—source data 2. [file elife-92426-fig3-data2.pdf]

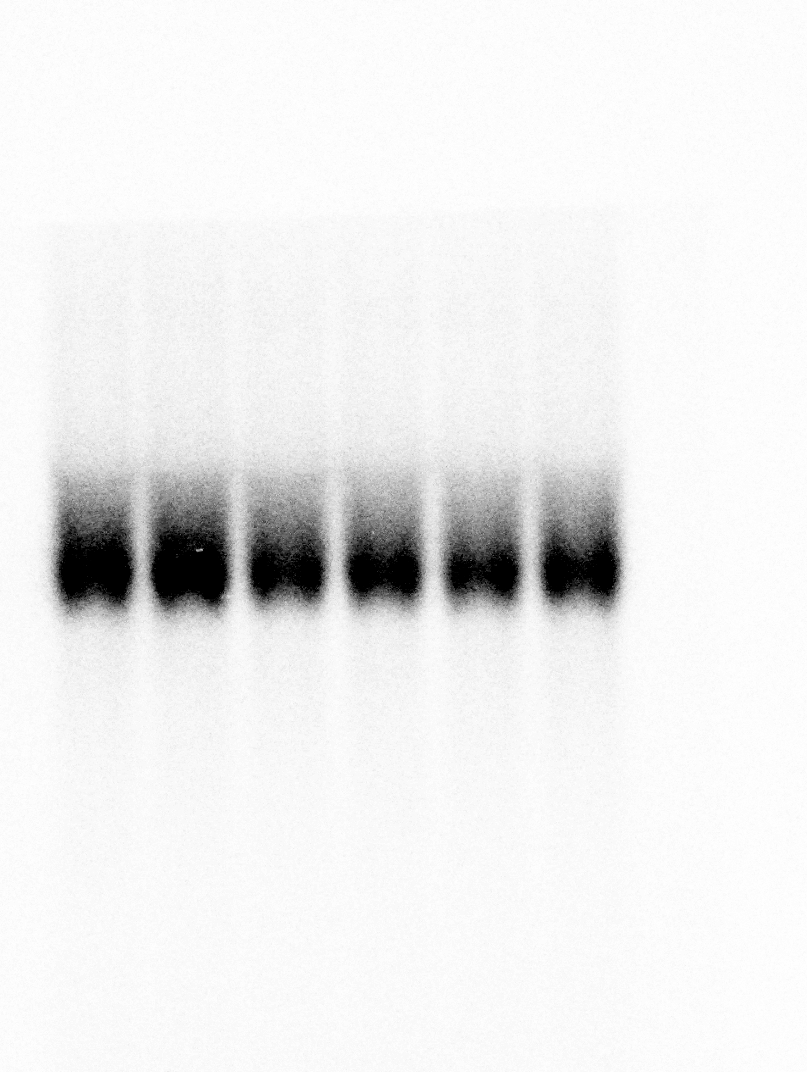

Supplement: Figure 4—source data 1. [file elife-92426-fig4-data1.zip › Figure 4-source data 1_Original file for the Northern blot in Figure 4B/DDX6ko-RL-AR.tif]

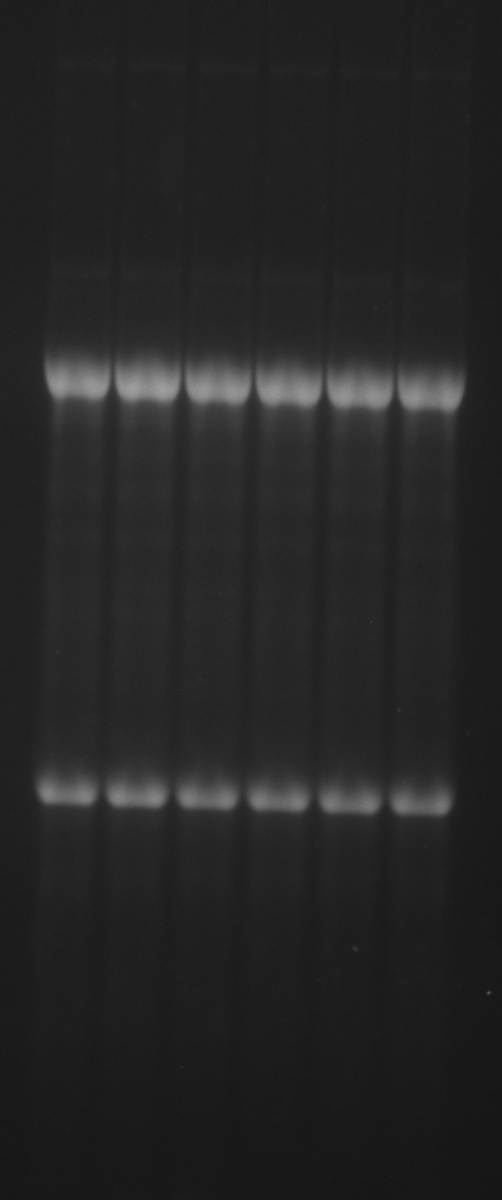

Supplement: Figure 4—source data 1. [file elife-92426-fig4-data1.zip › Figure 4-source data 1_Original file for the Northern blot in Figure 4B/DDX6ko-RL-AR_ribo.Tif]

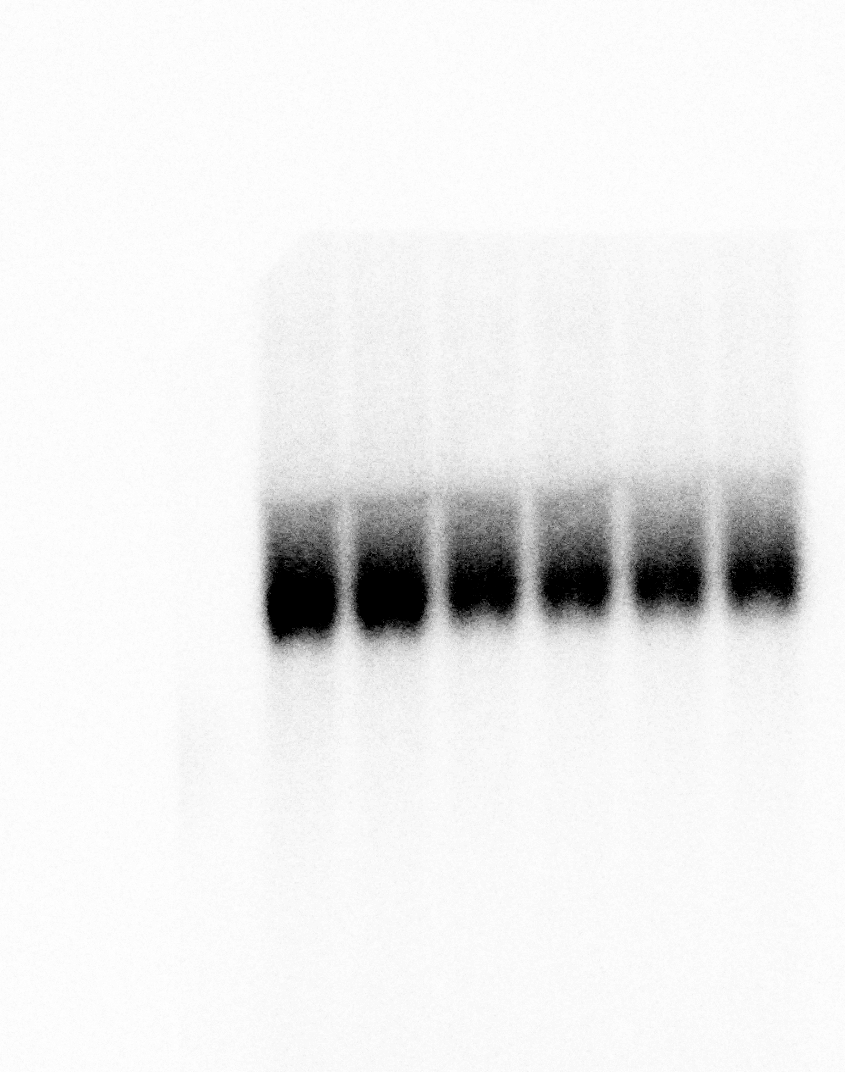

Supplement: Figure 4—source data 1. [file elife-92426-fig4-data1.zip › Figure 4-source data 1_Original file for the Northern blot in Figure 4B/DDX6ko-RL-stop-AR.tif]

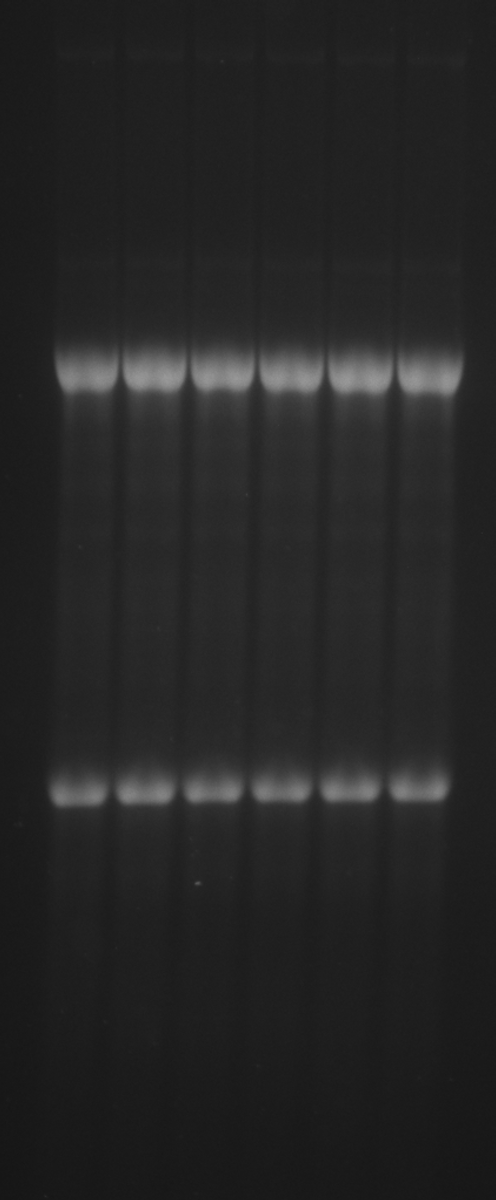

Supplement: Figure 4—source data 1. [file elife-92426-fig4-data1.zip › Figure 4-source data 1_Original file for the Northern blot in Figure 4B/DDX6ko-RL-stop-AR_ribo.tif]

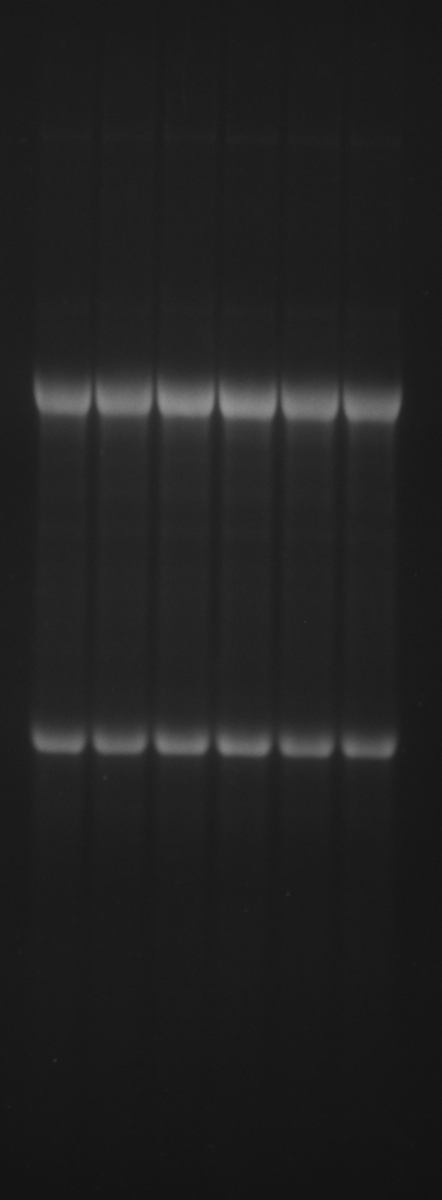

Supplement: Figure 4—source data 1. [file elife-92426-fig4-data1.zip › Figure 4-source data 1_Original file for the Northern blot in Figure 4B/WT-AR_ribo.tif]

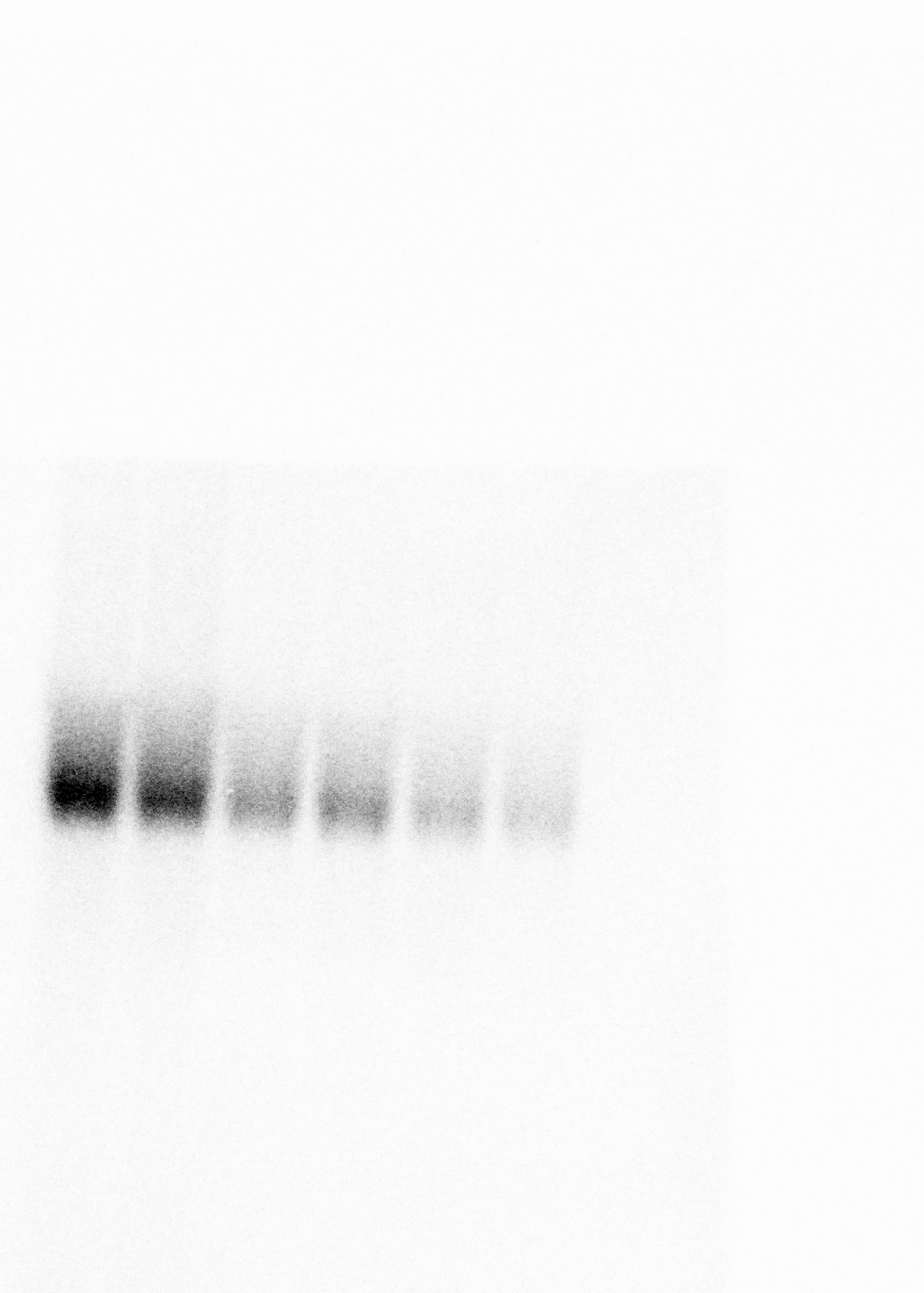

Supplement: Figure 4—source data 1. [file elife-92426-fig4-data1.zip › Figure 4-source data 1_Original file for the Northern blot in Figure 4B/WT-RL-AR.tif]

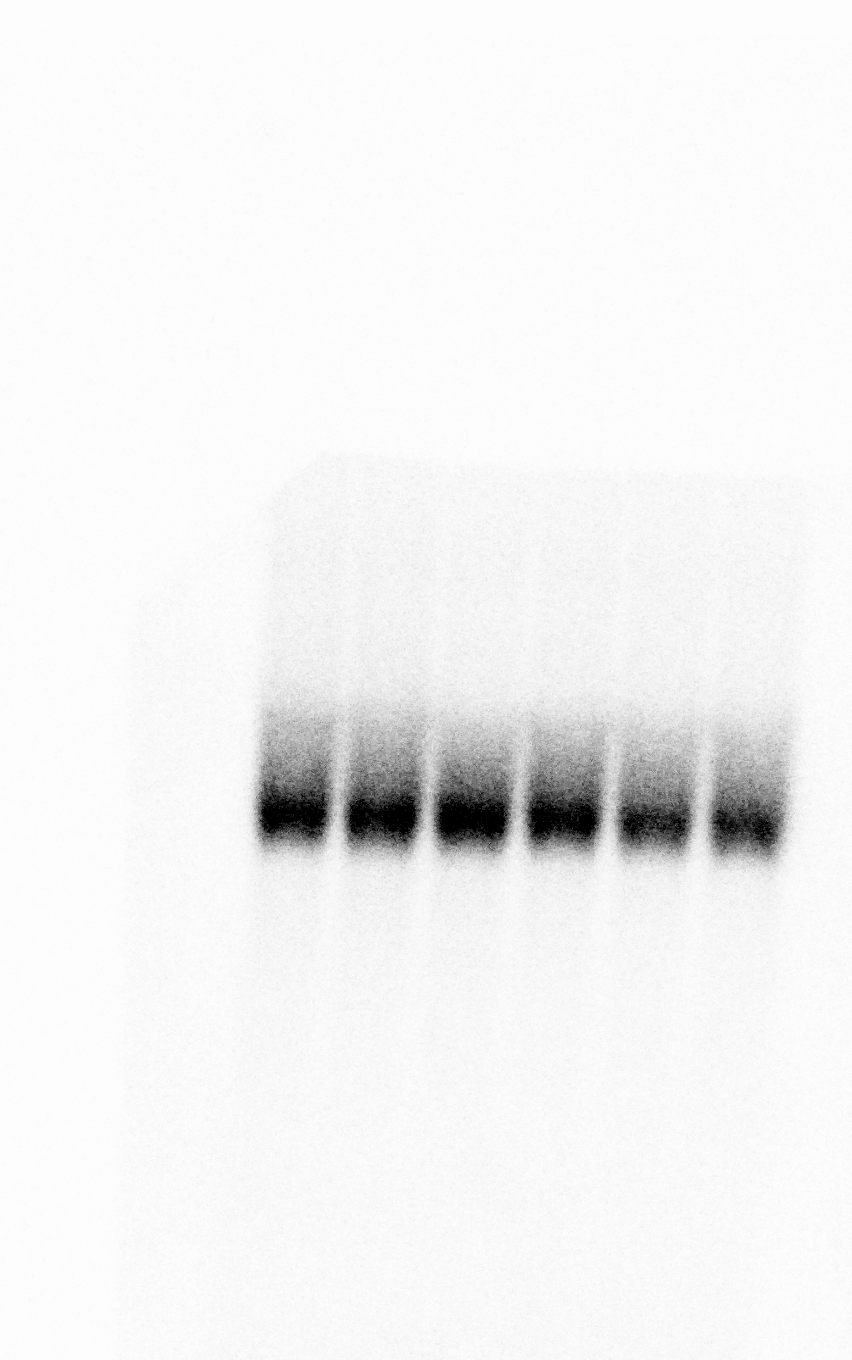

Supplement: Figure 4—source data 1. [file elife-92426-fig4-data1.zip › Figure 4-source data 1_Original file for the Northern blot in Figure 4B/WT-RL-stop-AR.tif]

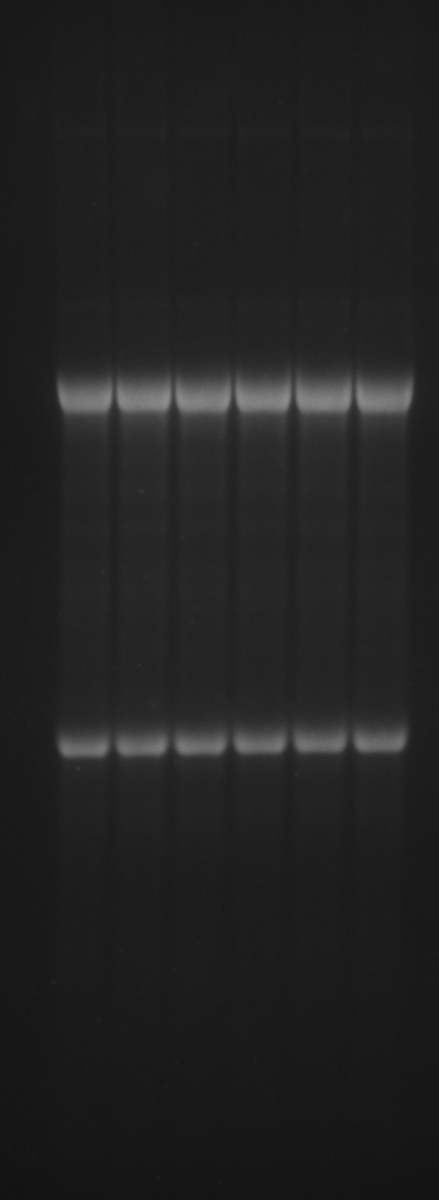

Supplement: Figure 4—source data 1. [file elife-92426-fig4-data1.zip › Figure 4-source data 1_Original file for the Northern blot in Figure 4B/WT-RL-stop-AR_ribo.tif]

Figure 4

**B** Ribosome stalling reporter AR mRNA

**B** Ribosome stalling reporter AR mRNA

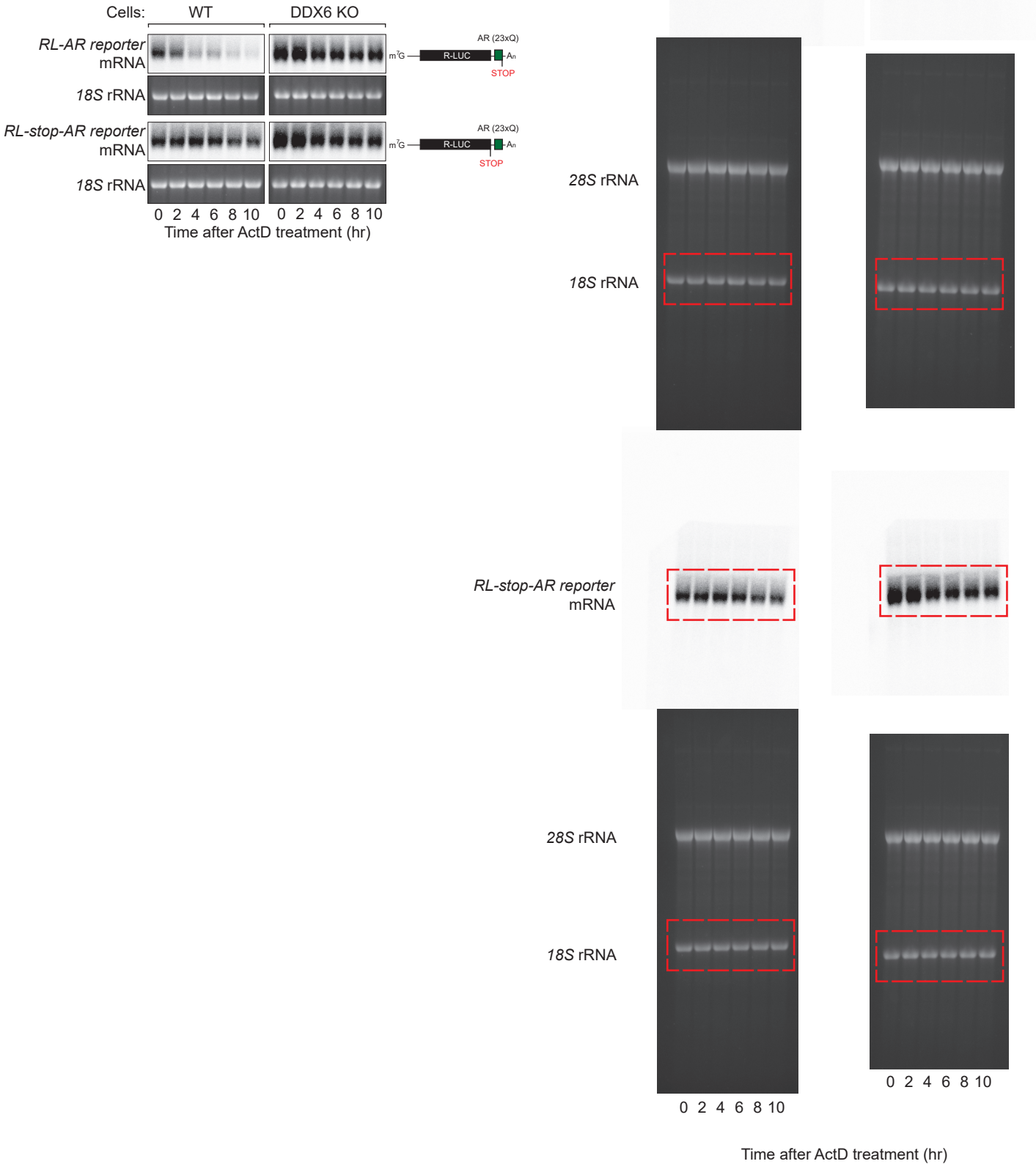

Supplement: Figure 4—source data 2. [file elife-92426-fig4-data2.pdf]

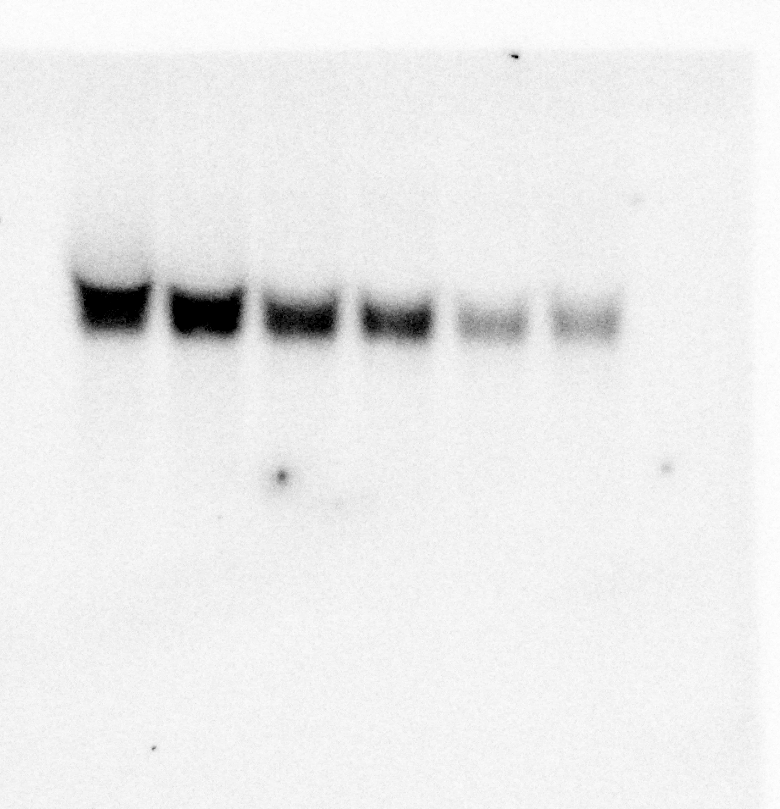

Supplement: Figure 4—source data 3. [file elife-92426-fig4-data3.zip › Figure 4-source data 3_Original file for the Northern blot in Figure 4D/DDX6ko-RL-BMP2.tif]

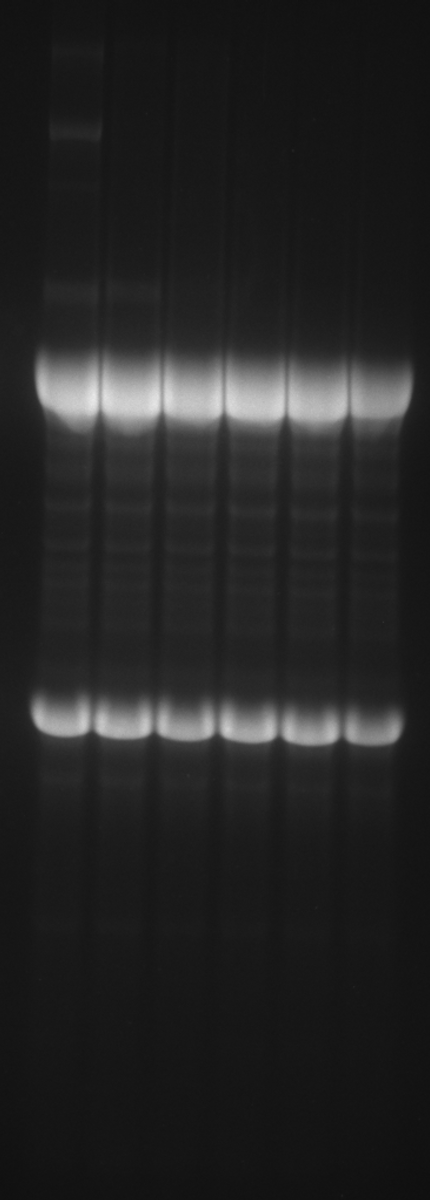

Supplement: Figure 4—source data 3. [file elife-92426-fig4-data3.zip › Figure 4-source data 3_Original file for the Northern blot in Figure 4D/DDX6ko-RL-BMP2_ribo.tif]

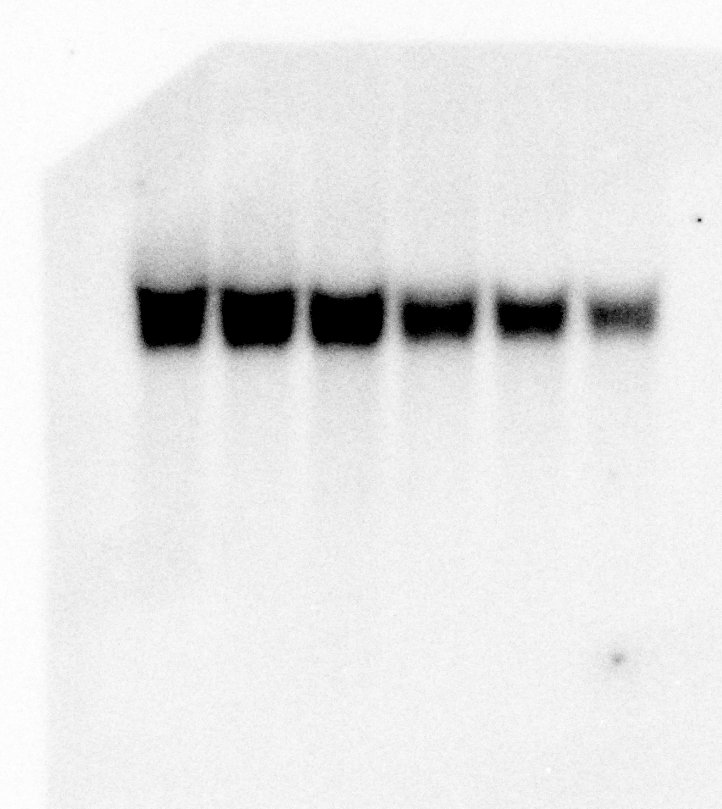

Supplement: Figure 4—source data 3. [file elife-92426-fig4-data3.zip › Figure 4-source data 3_Original file for the Northern blot in Figure 4D/DDX6ko-RL-stop-BMP2.tif]

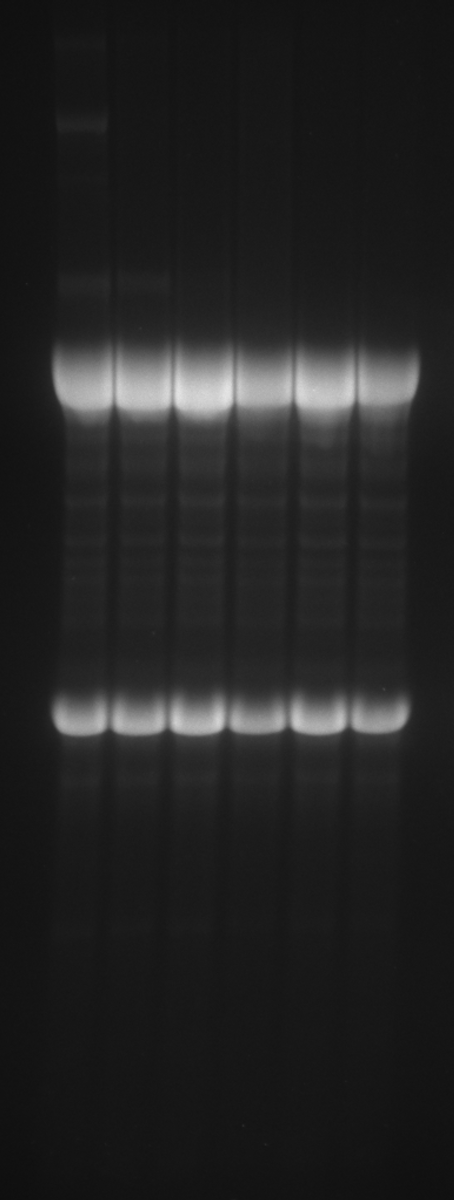

Supplement: Figure 4—source data 3. [file elife-92426-fig4-data3.zip › Figure 4-source data 3_Original file for the Northern blot in Figure 4D/DDX6ko-RL-stop-BMP2_ribo.tif]

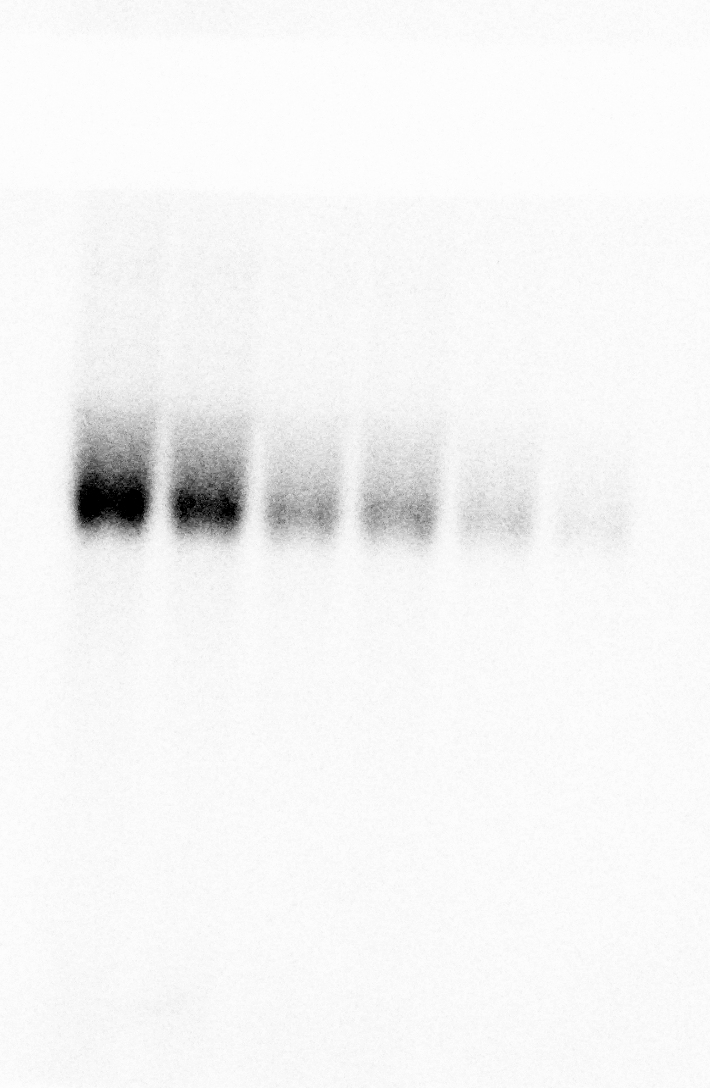

Supplement: Figure 4—source data 3. [file elife-92426-fig4-data3.zip › Figure 4-source data 3_Original file for the Northern blot in Figure 4D/WT-RL-BMP2.tif]

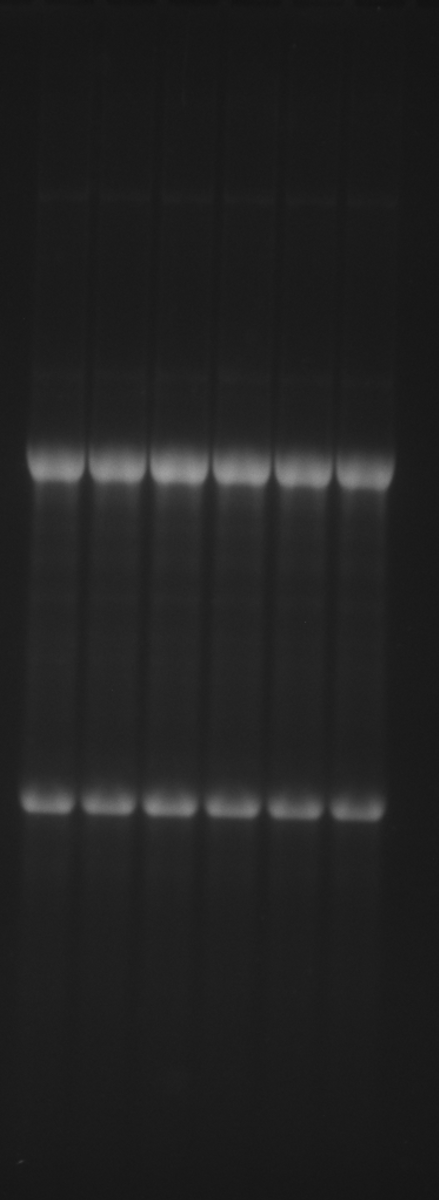

Supplement: Figure 4—source data 3. [file elife-92426-fig4-data3.zip › Figure 4-source data 3_Original file for the Northern blot in Figure 4D/WT-RL-BMP2_ribo.tif]

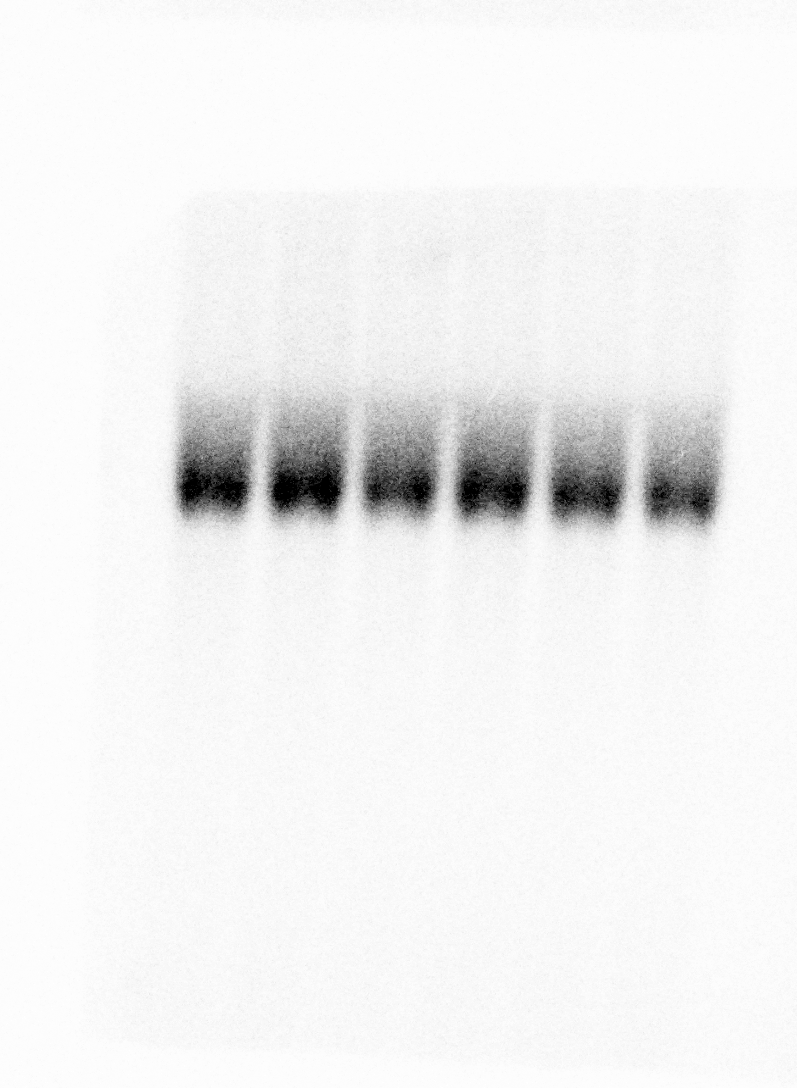

Supplement: Figure 4—source data 3. [file elife-92426-fig4-data3.zip › Figure 4-source data 3_Original file for the Northern blot in Figure 4D/WT-RL-stop-BMP2.tif]

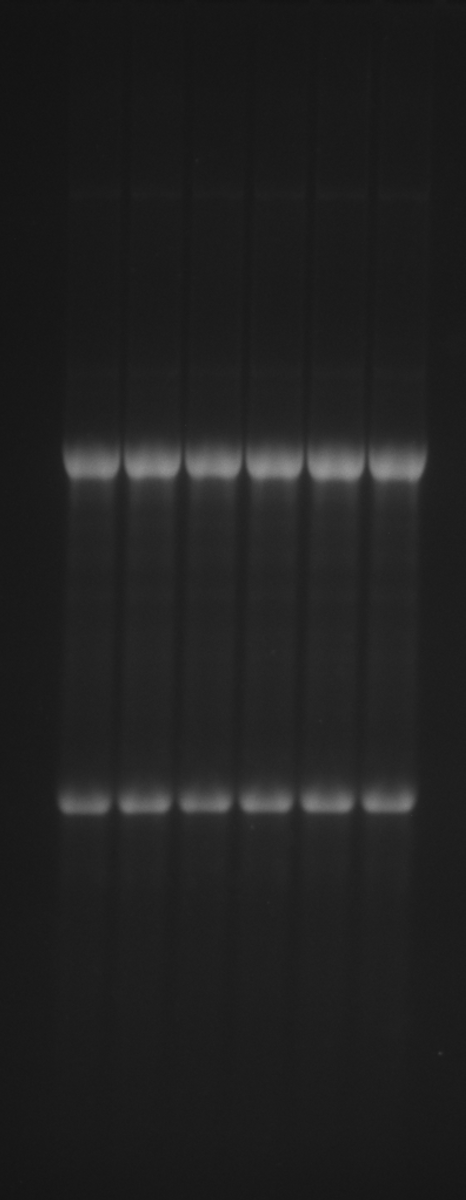

Supplement: Figure 4—source data 3. [file elife-92426-fig4-data3.zip › Figure 4-source data 3_Original file for the Northern blot in Figure 4D/WT-RL-stop-BMP2_ribo.tif]

Figure 4

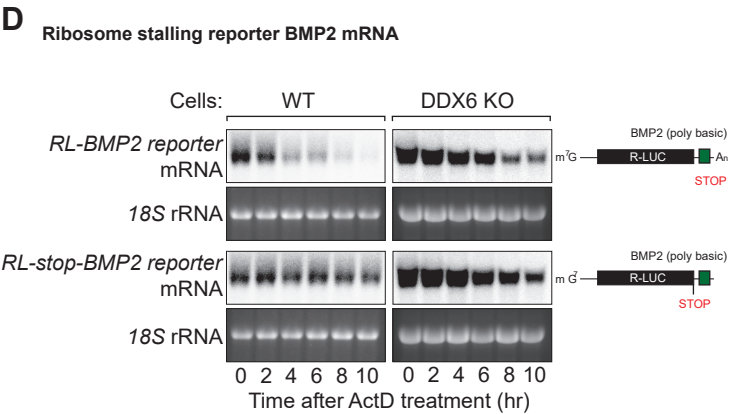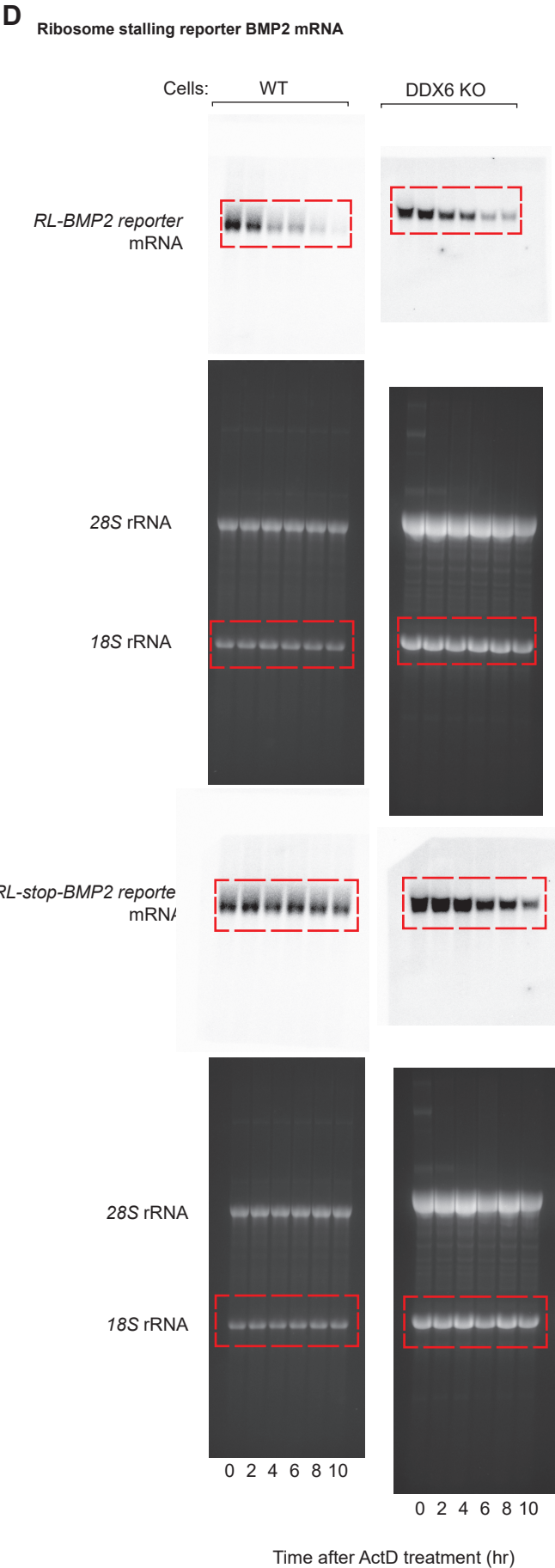

Supplement: Figure 4—source data 4. [file elife-92426-fig4-data4.pdf]

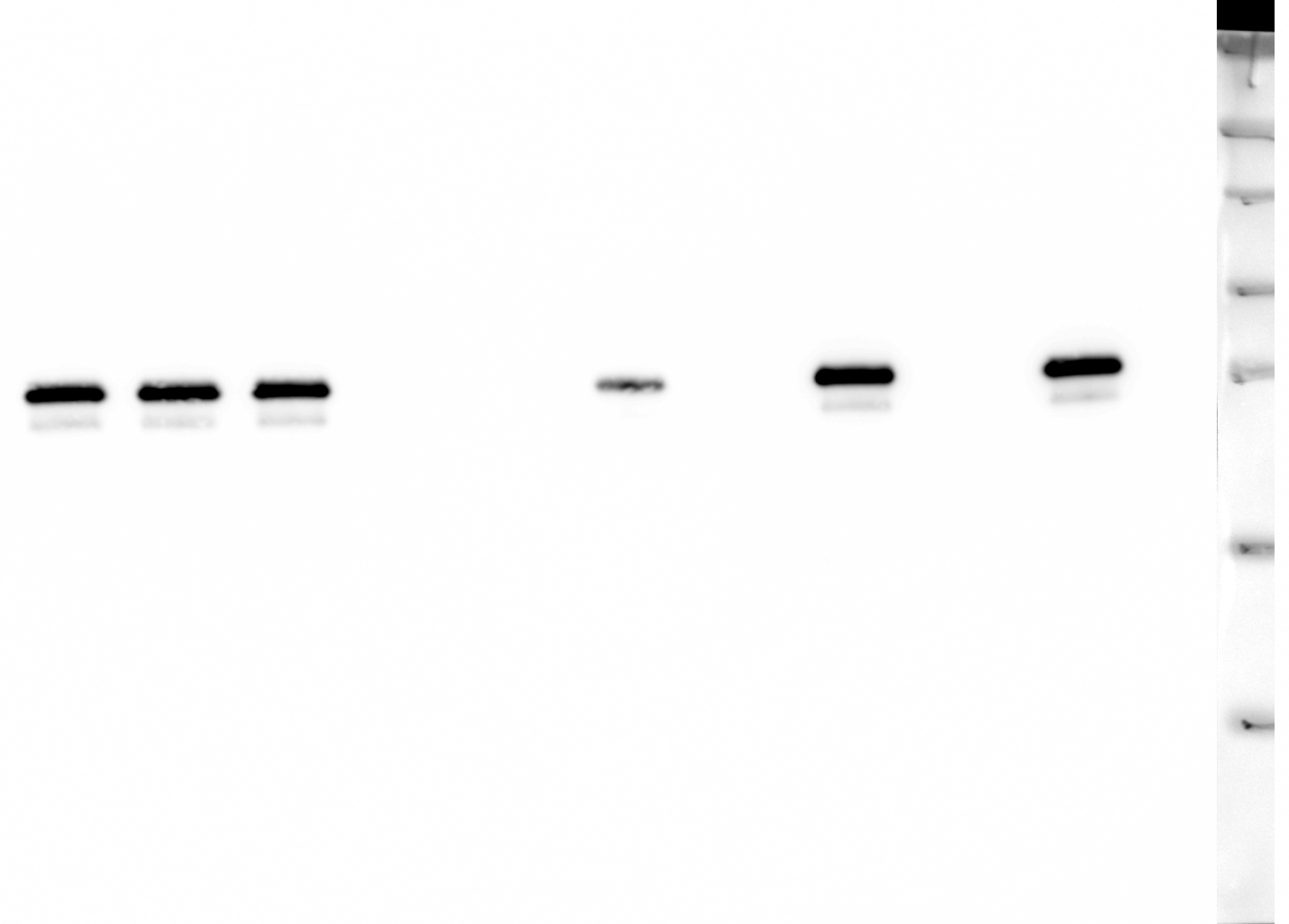

Supplement: Figure 4—source data 5. [file elife-92426-fig4-data5.zip › Figure 4-source data 5_Original file for the RNA IP analysis in Figure 4D/aHA-RPL22.tif]

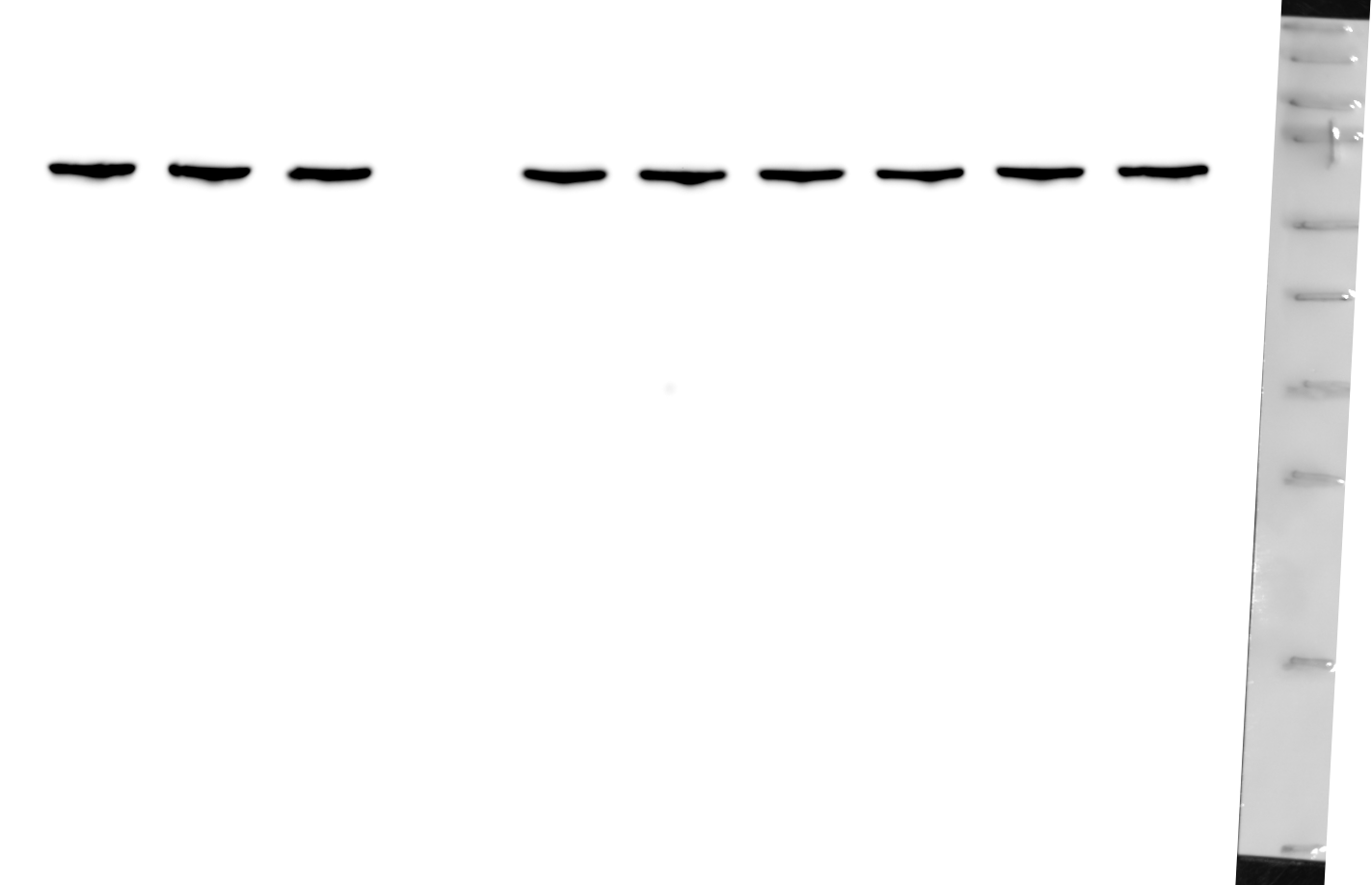

Supplement: Figure 4—source data 5. [file elife-92426-fig4-data5.zip › Figure 4-source data 5_Original file for the RNA IP analysis in Figure 4D/aV5-SBP-MBP-MS2.tif]

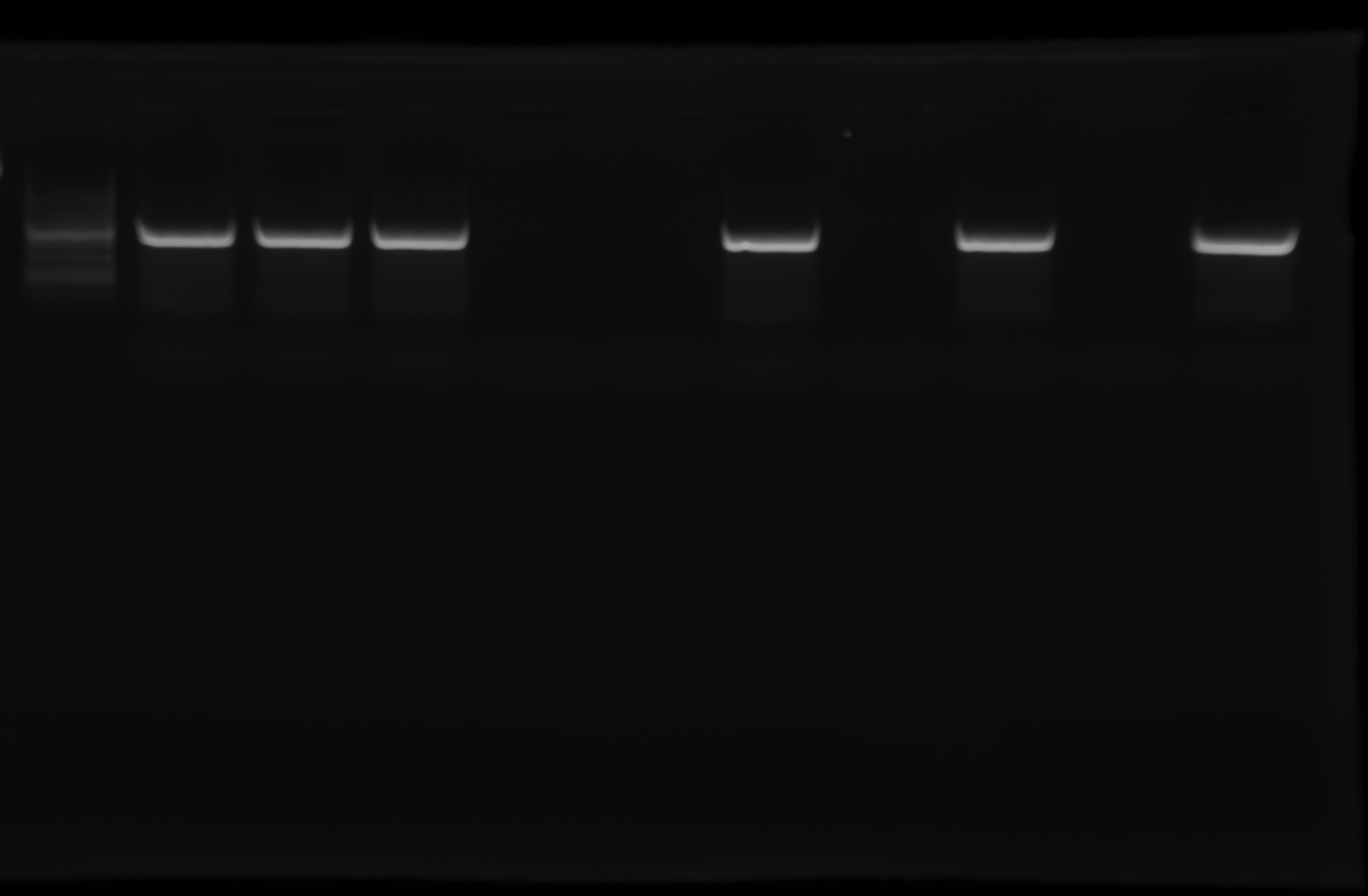

Supplement: Figure 4—source data 5. [file elife-92426-fig4-data5.zip › Figure 4-source data 5_Original file for the RNA IP analysis in Figure 4D/DSC00806_c.tif]

Figure 4

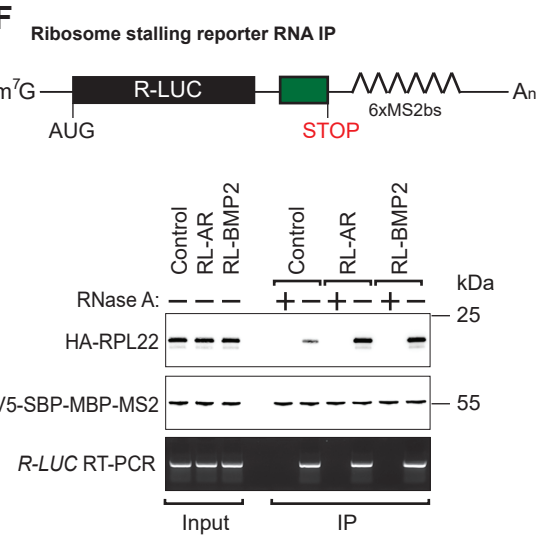

**F** Ribosome stalling reporter RNA IP

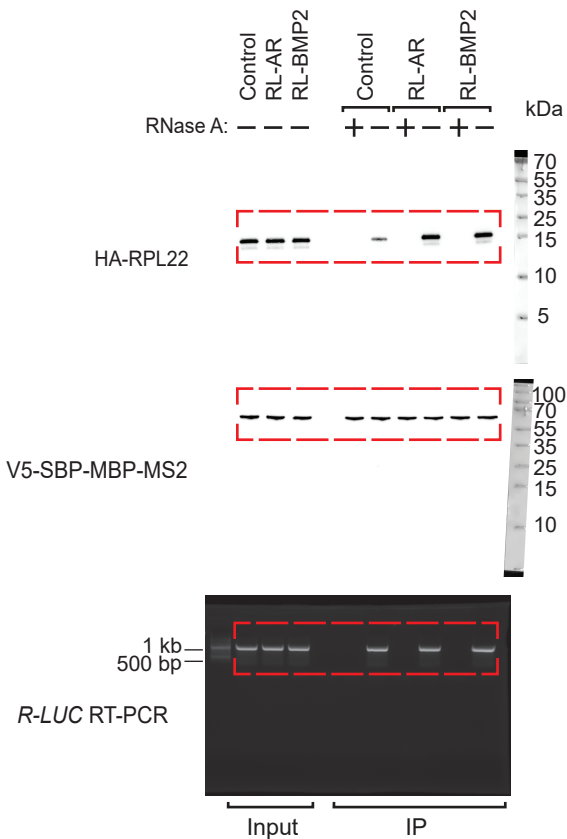

Supplement: Figure 4—source data 6. [file elife-92426-fig4-data6.pdf]
