# Supplementary material for: Human DDX6 regulates translation and decay of inefficiently translated mRNAs
Source: eLife. 2024 Jul 11;13:RP92426. doi: 10.7554/eLife.92426 (PMC11239181; doi:10.7554/eLife.92426)
Supplement: Supplementary file 1. [file elife-92426-supp1.docx]

**Supplementary File 1. Constructs and mutants used in this study.**

| **Name** | **Fragments / mutations** | **Plasmid** |
| --- | --- | --- |
| R-LUC | - | pCIneo-RLuc |
|  | *Last 30 codon sequence*  AAG GGC CTC CAC TTC AGC CAG GAG GAC GCT  CCA GAT GAA ATG GGT AAG TAC ATC AAG AGC  TTC GTG GAG CGC GTG CTG AAG AAC GAG CAG | |
| R-LUC 30xRC | 30xRC | pCIneo-RLuc_30xRC |
|  | *Last 30 codon sequence*  AAA GGT CTA CAT TTT TCG CAA GAA GAT GCG  CCG GAT GAA ATG GGT AAA TAT ATA AAA TCG  TTT GTA GAA CGT GTA CTA AAA AAT GAA CAA | |
| MBP-Strep | - | pnEK-NvHM-Strep-MBP |
| NusA-Strep-DDX6 | - | pETM-60-NusA-3C-HsRCK_296-472-Strep |
| GFP-MBP | - | pT7-EGFP-C1-MBP |
| GFP-DDX6 | - | pT7-EGFP-C1-HsDDX6 |
|  | N-ter | pT7-EGFP-C1-HsDDX6_1-295 |
|  | C-ter | pT7-EGFP-C1-HsDDX6_296-463 |
|  | E236Q | pT7-EGFP-C1-HsDDX6_E236Q |
|  | Mut1 | pT7-EGFP-C1-HsDDX6_Mut1 |
|  | Mut2 | pT7-EGFP-C1-HsDDX6_Mut2 |
| HA-RPL22 | - | pCIneo-HA-RPL22 |
| RL-AR | - | pCIneo-RL-AR |
| RL-stop-AR | - | pCIneo-RL-stop-AR |
| RL-BMP2 | - | pCIneo-RL-BMP2 |
| RL-stop-BMP2 | - | pCIneo-RL-stop-BMP2 |
| V5-SBP-MBP-MS2 | - | pCIneo-v5-SBP-MBP-MS2 |
| RL-6xMS2bs | - | pCIneo-RL-6xMS2bs |
| RL-AR-6xMS2bs | - | pCIneo-RL-AR-6xMS2bs |
| RL-BMP2-6xMS2bs | - | pCIneo-RL-BMP2-6xMS2bs |
